# Multi‐Instably Mechanical Systems for Computing‐Storing Functions

**DOI:** 10.1002/advs.202510880

**Published:** 2025-09-25

**Authors:** Jiajun Wang, Chenjie Zhang, Qianyun Zhang, Pengcheng Jiao

**Affiliations:** ^1^ State Key Laboratory of Ocean Sensing & Ocean College Zhejiang University Zhoushan Zhejiang 316021 China; ^2^ Hainan Institute Zhejiang University Sanya Hainan 572000 China; ^3^ Ocean College Zhejiang University Zhoushan 316021 China; ^4^ Department of Civil and Environmental Engineering New Mexico State University Las Cruces NM 88003 USA

**Keywords:** digital logic gates, fourth‐order ODE solvers, mechanical memories, multi‐instably mechanical systems

## Abstract

Computations based on mechanical systems exhibit unconventional environmental adaptability and interaction capabilities beyond electronical computing. In this research, the multi‐instably mechanical systems are proposed to achieve the functions of scientific computing, logical operations, and data storage, i.e., solving fourth‐order ordinary differential equations (ODEs) by ODE solvers, realizing logical operations by digital logic gates, and stably storing data by mechanical memories. The mechanical solvers obtain the special solutions for the fourth‐order ODEs by determining the unknow factors in the general solution via capturing the deformation shapes of multi‐instable beams, and the results are validated with the existed theoretical solutions with satisfactory agreements. The multi‐instable beams are then converted into the reconfigurable switches to build the digital logic gates, and assembled into 1 × 8 and 5 × 8 matrices for the mechanical memories. The reported computing‐storing system can accomplish interaction between the logical operation and storage modules. Eventually, the mechanical systems are discussed with the integrated functions that can perform scientific computing and logical operations in the computation module while mechanically saving the computing results in the storage module. The reported multi‐instably mechanical systems open a promising path to expand the mechanical computing from separate function to multifunctionality.

## Graphical abstract

Multi‐instably mechanical systems for the functions of scientific computing, logical operations, and data storage, i.e., solving fourth‐order ordinary differential equations (ODEs) by fourth‐order ODE solvers, realizing logical operations by digital logic gates, and stably storing data by mechanical memories. The reported multi‐instably mechanical systems open a promising path to expand the mechanical computing systems from separate function to multifunctionality.

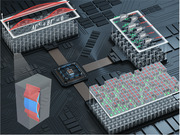

## 1 Introduction

Analog computing was originally implemented by mechanical mechanisms, and successfully applied to solve incredibly complicated questions such as fire‐control and bomb trajectory prediction over a long time in human history.^[^ ^1^ ^]^ Classic mechanical analog computers include knots, abacus, planimeters, harmonic synthesizers and analyzers in different forms.^[^ ^2^ ^]^ During the last century, electronic computers progressively replaced the mechanical analog computers owing to their superiorities in miniaturization and integration.^[^ ^3^ ^]^ Recently, an unconventional mechanical computing strategy was reported with the capacities of perceiving and processing the information from the environment beyond the electronic computing. Specific structural response or property, e.g., deformation state, stiffness or Poisson's ratio,^[^ ^4^ ^]^ is converted into information in the mechanical computing. Various mechanical computing systems have been developed to execute the information processing, e.g., logical operations^[^ ^5^, ^6^, ^7^, ^8^, ^9^, ^10^, ^11^, ^12^, ^13^, ^14^, ^15^, ^16^, ^17^, ^18^, ^19^, ^20^, ^21^ ^]^ and data storage,^[^ ^22^, ^23^, ^24^, ^25^, ^26^, ^27^, ^28^ ^]^ which are significant for the intelligent mechanical systems, such as robotic materials,^[^ ^29^, ^30^, ^31^, ^32^ ^]^ micro‐electro‐mechanical systems,^[^ ^33^ ^]^ and soft devices,^[^ ^34^, ^35^ ^]^ etc. For example, digital logic gates were accomplished by correlating the buckling modes and conductibility of cellular mechanical metamaterials made of conductive polymers.^[^ ^8^ ^]^ Stable and reprogrammable memory was reported by utilizing the magnetically actuated bistable units to construct a tiling mechanical metamaterial.^[^ ^23^ ^]^ Mechanical metamaterial electronics were developed to perform self‐powered, mechanoelectrical logical operations and information storage.^[^ ^36^ ^]^ However, the majority of existed mechanical computing systems can only perform the separate function of logical operations or data storage. In order to improve their scalability and applicability, it is of desire to expand the mechanical computing systems with separate function to the devices with multifunctionality of computing‐storing that combine a variety of computational functions.

**Figure 1:**
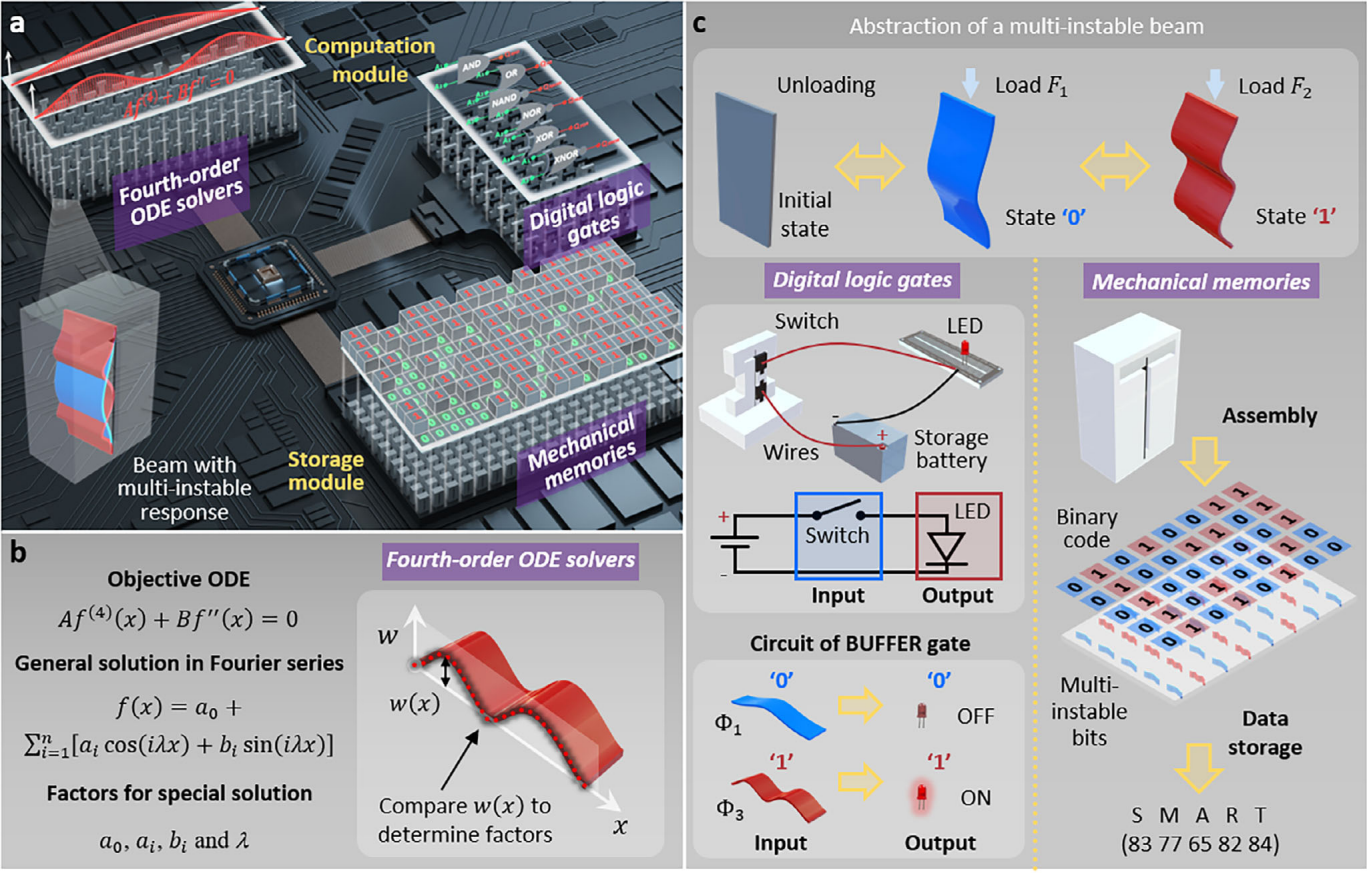
Principle of the multi‐instably mechanical systems. a) Multi‐instably mechanical systems with the functions of four‐order ordinary differential equation (ODE) solvers and digital logic gates in the computation module and mechanical memories in the storage module. b) Fourth‐order ODE solvers to obtain the special solutions of fourth‐order ODEs by determining the unknown factors via comparing with the deformation shapes of multi‐instable beams. c) Digital logic gates for realizing the logical operations using the bi‐walled beams with multi‐instable response as reconfigurable switches in circuits. Mechanical memories for stably storing information (i.e., ASCII characters and integral numbers) by assembling multi‐instable beam bits into matrices and converting the bits’ multi‐instable states into binary data.

To this end, we propose the multi‐instably mechanical systems that use the multi‐instable states of bilaterally constrained beams to realize the functions of scientific computing, logical operations and data storage. The fourth‐order ODE solvers and digital logic gates constitute the computation module to separately perform the scientific computing and logical operations, while the mechanical memories constitute the storage module to execute the data storage, as shown in **Figure** **1**a. In particular, the fourth‐order ODE solvers obtain the special solutions of fourth‐order ODEs by determining the unknown factors via comparing with the deformation shapes of the multi‐instable beams (Figure 1b). The digital logic gates and mechanical memories are implemented by converting the multi‐instable states of bilaterally constrained beams into binary data. The multi‐instable beams are converted into the reconfigurable switches to obtain logic operations of Buffer, NOT, AND, OR, NAND, NOR, XOR and XNOR, and then assembled into 1×8 and 5×8 matrices to stably store information (i.e., ASCII characters and integral numbers) (Figure 1c). The reported computing‐storing system can accomplish the interaction between the logical operation and storage modules. Eventually, we discuss the mechanical systems with the integrated functions that can store the results from the computation module (i.e., both fourth‐order ODE solvers and digital logic gates) in the storage module (i.e., mechanical memories) in real time. The multi‐instably mechanical systems open a promising path to expand the mechanical computing devices from separate function to multifunctionality of scientific computing, logical operations, and data storage.

## 2 Results

### 2.1 Mechanical Fourth‐Order ODE Solvers for Solutions of Fourth‐Order ODEs

Expanding mechanical solvers to scientific computing, we consider the fourth‐order ODEs in the general form of

(1) $$Af^{\left(4\right)}\left(x\right)+Bf^{\prime \prime }\left(x\right)=0$$

with the boundary conditions of

(2)

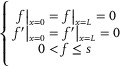

where *A*, *B*, *s* and *L* are the arbitrary input parameters, and *f*(*x*) is the objective solution. The general solution of Equation (1) can be written in the Fourier series with unknow factors as

(3) $$F\left(x\right)=a_{0}+\sum_{i=1}^{n}\left[a_{i}\cos\left(i\lambda x\right)+b_{i}\sin\left(i\lambda x\right)\right]$$

where *a*_0_, *a_i_*, *b_i_* and λ are the unknown factors that need to be determined to obtain the special solutions. The fourth‐order ODE in Equation (1) can be used to describe the multi‐instable response of a slender beam subjected to bilateral constraints.^[^ ^37^ ^]^ In this case, the parameters *A*, *B*, *s* and *L* are referring to the physical meanings of the bending stiffness *EI* of the beam, axial force, effective gap between the bi‐constraints and beam length, respectively. As a consequence, we compare the deformation shapes of the bilaterally constrained beams with Equation (3) to mechanically determine the unknown factors in the general solution, and thus, obtain the special solutions of the fourth‐order ODEs.

**Figure 2:**
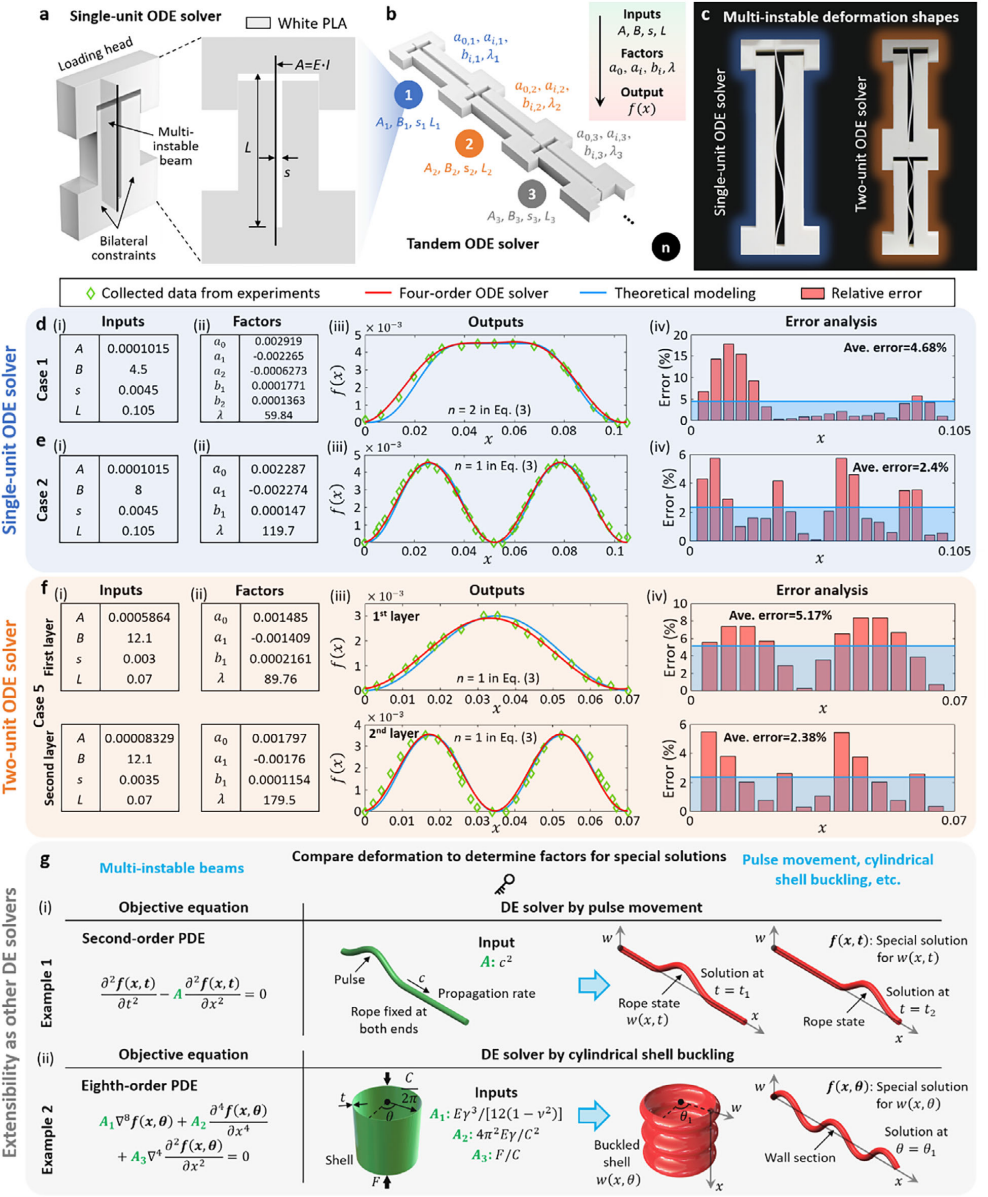
Mechanical fourth‐order ODE solvers by multi‐instable beams. a) Structural design of the single‐unit ODE solvers by multi‐instable beams subjected to bilateral constraints to solve the fourth‐order ODEs. b) Tandem ODE solvers for various fourth‐order ODEs with different input parameters by determining the unknown factors in the special solutions separately. c) Experimental deformation shapes of the single‐unit and two‐unit ODE solvers in the first and third buckling modes. Single‐unit ODE solver in the (d) first and (e) third buckling modes for: (i) input parameters in the fourth‐order ODE, (ii) unknown factors for the special solution, (iii) comparison between the theoretical and mechanical ODE output solutions and (iv) error analysis. f) Two‐unit ODE solver for: (i) input parameters, (ii) unknow factors, (iii) output solution comparison and (iv) error analysis in the first and second layers. g) Extensibility of the fourth‐order ODE solvers as other mechanical DE solvers, such as the DE solvers based on the pulse movement and cylindrical shell buckling for the PDEs.

**Figure** **2**a displays the structural design of the single‐unit ODE solvers to obtain the special solution *f*(*x*) by comparing with the deformations of multi‐instable beams under bi‐constraints. It is worthwhile pointing out that for the lower‐mode multi‐stable response (e.g., first and third buckling modes Φ_1_ and Φ_3_ in Figure 1c), Equation (3) typically does not need to expand more than five terms (i.e., *n* ≤ 5). In the same manner, various fourth‐order ODEs with different *A*, *B*, *s* and *L* can be simultaneously solved by the tandem ODE solvers consisted of a series of single‐unit ODE solvers, as shown in Figure 2b. We fabricated and experimentally tested the single‐unit and two‐unit ODE solvers by 3D printing using the material of polylactic acid (PLA) (Experimental Section for the fabrication and testing in detail). Figure 2c shows the experimental deformation shapes of the single‐unit and two‐unit ODE solvers in the first and third buckling modes. To validate the accuracy and feasibility, we compare the mechanical fourth‐order ODE solvers with the existing theoretical solutions. Figure 2d(i) presents the input parameters of the fourth‐order ODE and Figure 2d(ii) shows the factors for the special solution in Equation (3) for the case of single‐unit ODE solver in the first buckling mode Φ_1_. Figure 2d(iii) compares the special solution determined by the mechanical fourth‐order ODE solver with the existing theoretical solution. Such structurally multi‐stable problem has been well analyzed in the literature to obtain the theoretical solution of the shape function *w*(*x*) (see Equations ((4), (5), (6)) in Experimental Section). The deformation shape of multi‐stable beam of single‐unit ODE solver in experiments is used to determine the unknown factors in the special solution. Figure 2d(iv) displays the error analysis with an average error of 4.68% for the single‐unit ODE solver, where the relative error between the theoretical and Fourier series solutions is defined as |*f*(*x*) − *w*(*x*)|/*s*.

Figure 2e(i,ii) shows the input parameters of the fourth‐order ODE and the factors for the special solution for the case of single‐unit ODE solver in the third buckling mode Φ_3_, respectively. The result comparison and error analysis with an average error of 2.4% are shown in Figure 2e(iii,iv). Next, Figure 2f demonstrates the input parameters, unknow factors, output solution comparison and error analysis for the case of two‐unit ODE solver. As the ODE solvers in the first and second layers are independently connected, the input parameters and unknown factors are given separately. The theoretical and mechanical ODE solutions are separately compared with the average errors of 5.17% and 2.38% for the two layers in Φ_1_ and Φ_3_, respectively. Following the same assembly strategy, the tandem ODE solvers can be obtained to solve various fourth‐order ODEs with well accuracy at the same time. The structural and material parameters of the multi‐instable beams, loading and boundary conditions, quantity of collected coordinates, and expressions of the output solutions are given in Note S3 (Supporting Information). Shape extraction is carried out to accurately capture the configurations of the deformed beams (see Experimental Section).

The fourth‐order ODE solvers provide an easy‐to‐understand, easy‐to‐operate, and intuitive mechanical solution method for fourth‐order ODEs. In addition, this study proposes the tandem ODE solvers integrated by multiple single‐unit ODE solvers, which can simultaneously and efficiently solve the fourth‐order ODEs with different coefficients and boundary conditions, and intuitively compare the characteristics of their special solutions. More importantly, the proposed fourth‐order ODE solvers provide a promising strategy for solving differential equations (DEs), i.e., capturing the physical deformations of mechanical structures as solutions of specific DEs. As a consequence, the reported fourth‐order ODE solvers possess well scalability, which can be expanded to other types of DE solvers for mathematically difficult‐to‐solve DEs. For example, Figure 2g introduces the mechanical DE solvers based on the pulse movement and cylindrical shell buckling. Figure 2g(i) shows the forming principle of mechanical DE solver by the pulse movement for solving the objective equation of second‐order partial deferential equation (PDE). It can be seen that the rope with both ends clamped serves as the prototype of mechanical DE solver. Determining the pulse propagation rate on the rope by the coefficient of object equation, and the special solutions of objective equation can be obtained by recording the shape functions of rope at different times. Similarly, Figure 2g(II) shows the forming principle of mechanical DE solvers by the cylindrical shell buckling for solving the eighth‐order PDE. In particular, the cylindrical shell is used as the prototype of mechanical DE solver. The material and structural parameters, and applied load are provided by the coefficients of object equation. The special solutions of objective equation are given by capturing the shape functions of wall section of buckled shell.

### 2.2 Digital Logic Gates for Logical Operations

Here, we first develop the reconfigurable logic switches based on the multi‐instable response of the beam under composite bilateral constraints. The switches are designed with the conductive beam made of carbon black PLA (PLA‐CB), and composite bi‐constraints that have the conductive components made of PLA‐CB and nonconductive components made PLA, as shown in **Figure** **3**a.

Figure 3Digital logic gates by the multi‐instable beams for logical operations. a) Design of the reconfigurable composite switches 1 and 2 to form the mechanical input modules of the digital logic gates. b) SEM images for the microstructures of the 3D printing PLA‐CB and PLA. c) Abstraction of the multi‐instable responses of Φ_1_ and Φ_3_ into the mechanical inputs of A  =  0 and A  =  1, respectively. d) Switches 1 and 2 in the states of “Closed” and “Open” by letting the deformed conductive beams contact the conductive components of the constraints in different mechanical inputs. Circuit diagrams and experimental demonstrations for the digital logic gates with the single mechanical input of (e) Buffer and (f) NOT. Circuit diagrams for the four digital logic gates with double mechanical inputs of (g) AND, (h) NAND, (i) XOR, (j) XOR, and (k) combinatorial logic of half adder. l) Truth tables and (m‐q) experimental demonstrations of four digital logic gates with double mechanical inputs and half adder.

**Figure:**
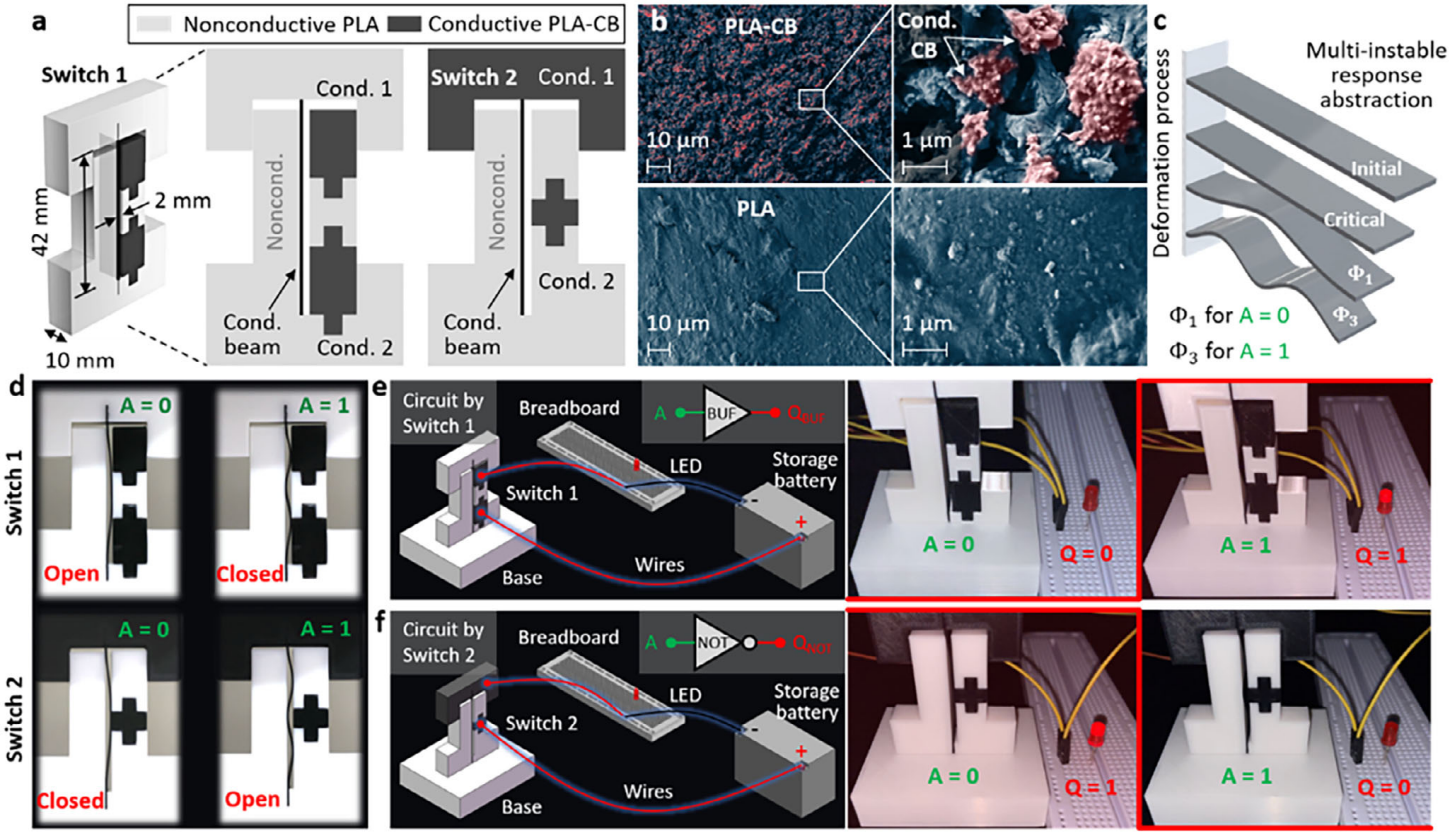

**Figure:**
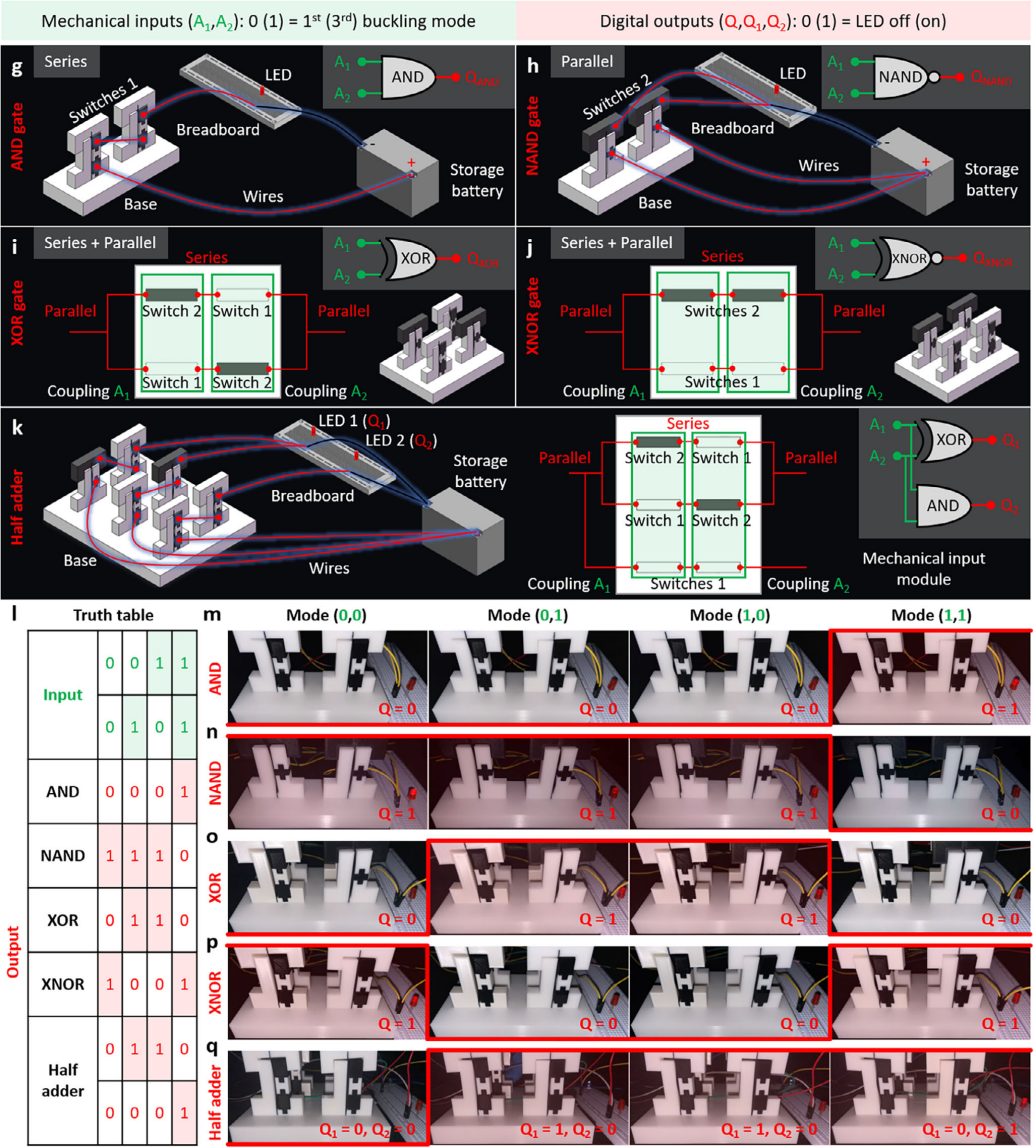

Figure 3b demonstrates the scanning electron microscope (SEM) images of the microstructures of the 3D printing PLA‐CB and PLA. The micromorphology of PLA‐CB filaments is rough and porous due to the mixture of carbon black, while the PLA filaments are smooth and dense. We abstract the multi‐instable responses Φ_1_ and Φ_3_ of the conductive beam into the mechanical binary digits (bits) of A  =  0 and A  =  1, respectively (Figure 3c). We turn the switches to the state of “Closed” by letting the deformed conductive beams contact the conductive components 1 and 2 (e.g., A  =  1 for switch 1 and A  =  0 for switch 2 in Figure 3d). The switches are kept as “Open” when the beams contact the nonconductive components (e.g., A  =  0 for switch 1 and A  =  1 for switch 2). We then build the digital logic gates by connecting the switches to a power supply and LED light in the circuits. The states of LED light serve as the digital outputs, i.e., LED OFF (ON) represents the digital outputs of Q  =  0 (Q  =  1). Figure 3e,f demonstrate the circuit diagrams and experimental demonstrations for the digital logic gates with the single mechanical input. In particular, the logic gates of Buffer and NOT are formed by using the switches 1 and 2 as the mechanical inputs, respectively. The Buffer gate leads to the digital output of Q  =  0 (i.e., LED is OFF) when the mechanical input is A  =  0 (i.e., the conductive beam is in Φ_1_), and the LED is ON (i.e., Q  =  1) when the conductive beam is in Φ_3_ (i.e., A  =  1). On the contrary, the NOT gate experiences LED ON in Φ_1_ and Q  =  0 when A  =  1. See Experimental Section for the fabrication and testing of the digital logic gates in detail. Note S4 (Supporting Information) presents the multi‐instable response of the logic switches.

The digital logic gates with double mechanical inputs (i.e., AND, OR, NAND, NOR, XOR and XNOR) can be achieved by exploiting the combinations of switches to construct the mechanical input modules. Figure 3g presents the circuit diagram of AND gate with the mechanical input module of two switches 1 in series, and Figure 3h shows the NAND gate by two switches 2 in parallel. The OR and NOR gates are formed, respectively, by connecting the mechanical input modules of two switches 1 in parallel and two switches 2 in series, as shown in Note S5 (Supporting Information). Different from the above gates, the XOR and XNOR gates are designed with two switches 1 and two switches 2 to build the mechanical input modules. The constitutions of the coupling mechanical inputs A_1_ and A_2_ and the connections of the switches in the XOR and XNOR gates are demonstrated in Figure 3i,j. Further integrating the eight digital logic gates, complicated logical operations can be implemented. Figure 3k shows the circuit diagram of the combinatorial logic of half adder constituted of the AND and XOR gates. Figure 3l shows the truth tables of AND, NAND, XOR, XNOR and half adder, and Figure 3m–q experimentally display their logical operations. For example, the AND gate with the digital output Q  =  1 (i.e., LED is ON) exclusively occurs when the mechanical inputs are A_1_ = 1 and A_2_ = 1, as shown in Figure 3m. Figure 3n presents the NAND gate with the LED is ON, which is obtained by leastwise one of the switches is in Φ_1_ (i.e., A_1_ = 0 or A_2_ = 0). However, at least one branch of mechanical input module needs to be conductive to keep the LED ON (i.e., Q  =  1) in the circuit diagrams of the XOR and XNOR gates. The experimental demonstrations of eight digital logic gates and half adder are provided in detail in Note S6 (Supporting Information).

### 2.3 Mechanical Memories for Stable Data Storage

**Figure 4:**
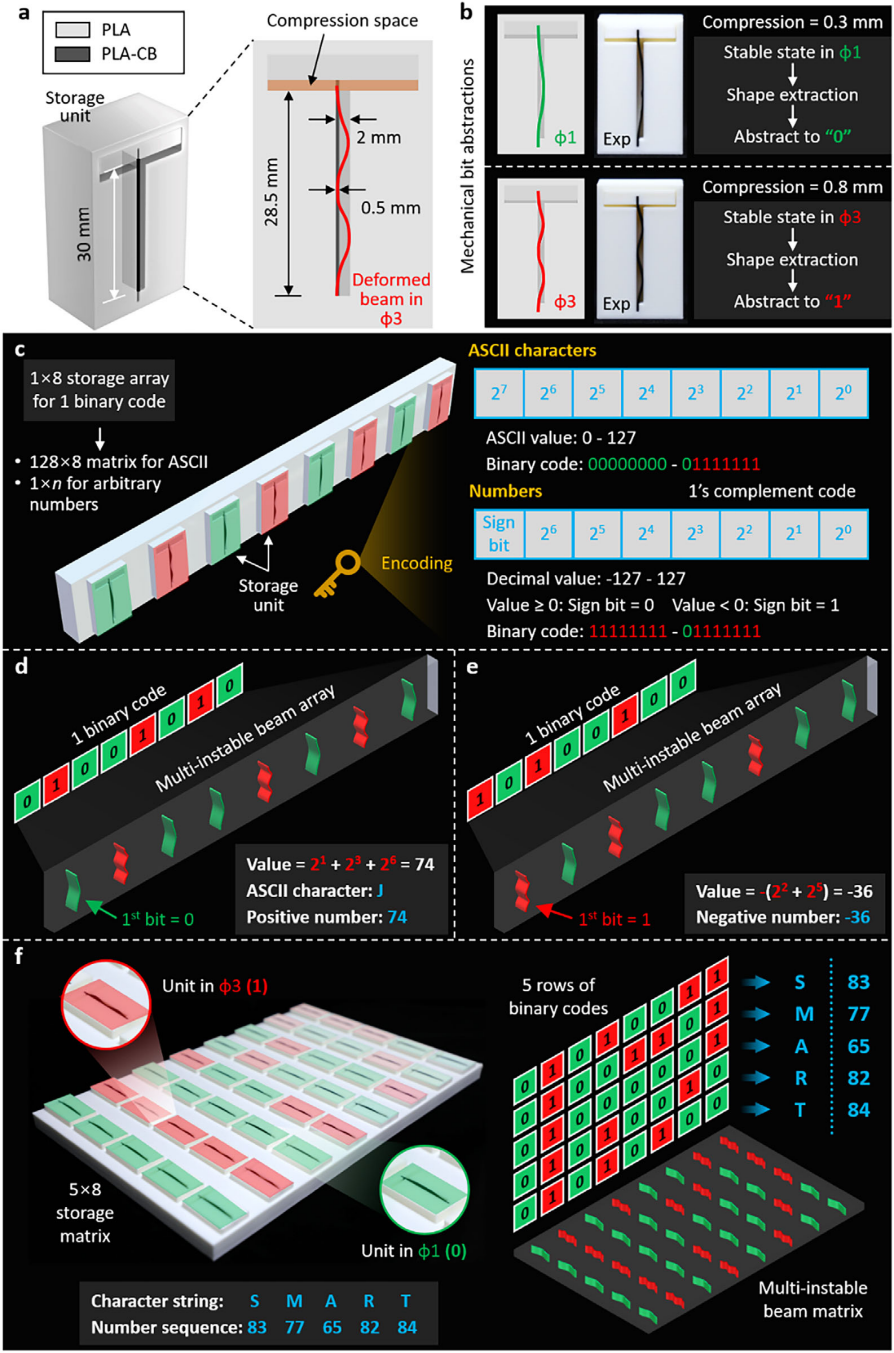
Mechanical systems by multi‐instable beams for mechanical memories. a) Storage unit designed in the fixed frame with compression space for stable deformation shapes of Φ_1_ and Φ_3_. b) Abstraction of mechanical bits from stable deformation states to binary bits of “0” and “1” by shape extraction. c) Assembling eight storage units into a 1 × 8 storage array, which leads to the strategy of combining 128 × 8 units to cover the entire ASCII table ordered from 0–127 or grouping an 1 × *n* storage array, n∈[1,∞) to save any decimal numbers. d) Binary code and multi‐instable beam array that are overlapped for the ASCII character of “J” and decimal number of “74”, and (e) binary code and multi‐instable beam array that are only for the decimal number of “‐36″. f) Experimental demonstration of the 5×8 storage matrix for storing the character string of “S”, “M”, “A”, “R”, and “T”, and the number sequence of “83”, “77”, “65”, “82”, and “84”.

The essential of building the mechanical memories is to convert the volatile multi‐instable beam deformations (i.e., Φ_1_ and Φ_3_ in Figure 1c) into stable states such that to maintain the data storage. To this end, we embed the bilateral constraints into the fixed frame with an adjustable compression space, where the inside length of 28.5 mm is smaller than the beam length of 30 mm. This leads to the condition that the multi‐instable beam is forced to be in the deformation shapes of Φ_1_ and Φ_3_, as shown in **Figure** **4**a. To ensure that the beam deformations occur as expected, we conducted the compression testing of the bi‐constrained beam to accurately design the structural parameters of the storage unit (see Note S7, Supporting Information). Figure 4b demonstrates the mechanical bit abstractions from the stable states of Φ_1_ and Φ_3_ to “0” and “1” by shape extraction.

Assembling eight storage units into a 1×8 storage array, we obtain one binary code and accordingly, a 128×8 storage matrix completely covers the entire ASCII table ordered from 0–127. In addition, adding or removing the storage units to *n*, n∈[1,∞), a 1 × *n* storage array can store any decimal numbers. As a consequence, the binary codes are overlapped for the ASCII characters and decimal numbers ranged between 0 and 127. In this case, adding a category unit at the end of the array can accurately distinguish the stored information between characters and numbers. To store characters, the binary code range of “00000000” – “01111111” refers to the ASCII characters ordered from 0–127. To store numbers by the 1×8 array, the first unit signifies the sign bit of the integers, i.e., “0” (“1”) for positive (negative), so the binary code range of “11111111” – “01111111” covers the decimal values of ‐127–127. Figure 4c showcases the encodings of the ASCII characters and integral numbers by the 1×8 storage array. Figure 4d presents the ASCII character of “J” and integral number of “74” that can be identically saved by “01001010” (i.e., within the range of 0–127); on the contrary, Figure 4e shows the integral number of “‐36” that does not have an identical ASCII character. To demonstrate the expansion of the 1×8 storage array for more complicated information, Figure 4f presents the 5×8 storage matrix that was experimentally fabricated to store the ASCII character string of “S”, “M”, “A”, “R” and “T” and the number sequence of “83”, “77”, “65”, “82” and “84”.

### 2.4 Mechanical Systems for Computing‐Storing Functions

**Figure 5:**
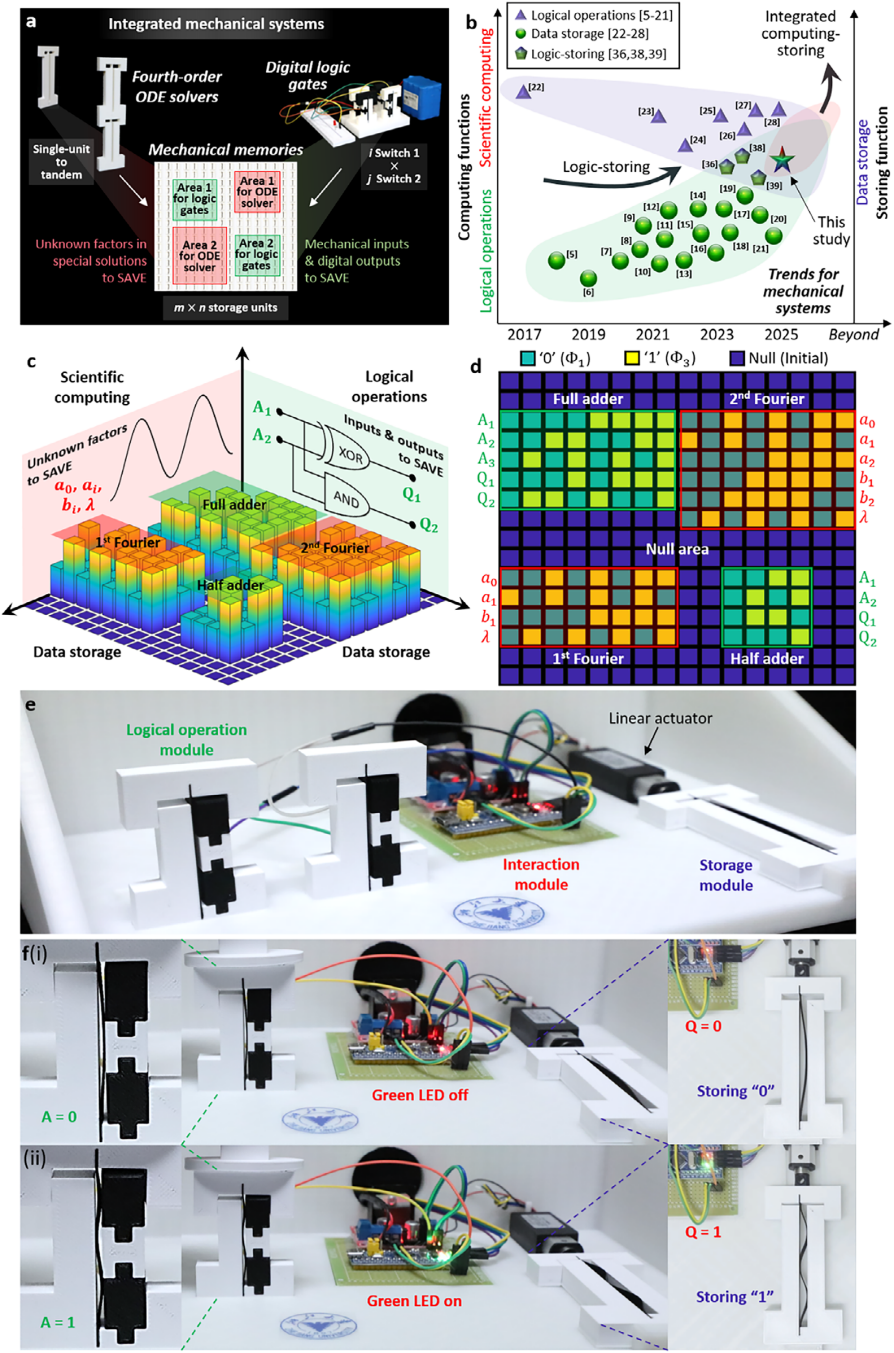
Mechanical systems with the functions of scientific computing, logic operations, and data storage. a) Principle of the mechanical systems that incorporate the computation module of fourth‐order ODE solvers and digital logic gates and the storage module of mechanical memories. b) Current status and research trends of mechanical devices from separate function to multifunctional integrated systems.^[^ ^5^, ^6^, ^7^, ^8^, ^9^, ^10^, ^11^, ^12^, ^13^, ^14^, ^15^, ^16^, ^17^, ^18^, ^19^, ^20^, ^21^, ^22^, ^23^, ^24^, ^25^, ^26^, ^27^, ^28^, ^36^, ^38^, ^39^ ^]^ c) Coordinated operations of the mechanical systems by simply storing the unknown factors (*a*_0_, *a_i_*, *b_i_* and λ) of the special solutions for the fourth‐order ODE solvers, and the mechanical inputs A_n_ and digital outputs Q_n_ for the digital logic gates. d) Exemplification of the mechanical systems in a patterned binary matrix that save the unknow factors and the inputs and outputs in the form of binary codes. e) Computing‐storing system constituted of the logical operation, storage, and interaction modules. f) Experimental demonstrations of the integrated system for the computing‐storing interactions.

Here, we demonstrate the mechanical systems with computing‐storing functions by incorporating the computation modules of fourth‐order ODE solvers and digital logic gates and the storage module of mechanical memories. The fourth‐order ODE solvers and digital logic gates separately perform the scientific computing and logical operations while the mechanical memories save the computing results, as shown in **Figure** **5**a. Figure 5b compares the recent studies and discusses the research trends of mechanical systems from separate function to multifunctional integrated systems.^[^ ^5^, ^6^, ^7^, ^8^, ^9^, ^10^, ^11^, ^12^, ^13^, ^14^, ^15^, ^16^, ^17^, ^18^, ^19^, ^20^, ^21^, ^22^, ^23^, ^24^, ^25^, ^26^, ^27^, ^28^, ^36^, ^38^, ^39^ ^]^ Functions of mechanical logical operations and data storage developed in their separate domains ever since the debut; however, have been experiencing the trend of integration during the last several years. For instance, the in‐memory computing system was reported with the computing‐storing functions by a network of non‐volatile binary mechanical memory units (i.e., buckled beams),^[^ ^38^ ^]^ and the sequential logic‐based material design framework was developed by integrating the nonvolatile memory with continuous processing.^[^ ^39^ ^]^ As a consequence, next‐generation mechanical systems can possess the integrated functions of scientific computing, logical operations and data storage. In order to achieve the ability of adapting behavior according to the retained knowledge (i.e., intelligence), intelligent mechanical systems such as robotic materials, micro‐electro‐mechanical systems and soft devices require a computation module for data processing and system adapting, a storage module for data storage, and an interaction module for communication between the computation and storage module.^[^ ^40^ ^]^ However, although mechanical computing devices offer the advantages of immunity to electromagnetic interference, data stability and data security compared to electronic computing, the lack of interaction module limits the performance of mechanical computing devices.^[^ ^38^ ^]^ Therefore, it is crucial to build up the computing‐storing mechanical systems in which computation and storage modules can interact with each other, so as to improve the compatibility with other mechanical components and realize intelligent mechanical systems.

The key to implementing computing‐storage mechanical systems is the coordinated operation of computation and storage modules, i.e., storing the results from the computation module in real time by the storage module. Figure 5c presents the conceptual schematic of coordinated operation of mechanical systems. To save space and improve efficiency in the 16×16 storage matrix, we only store the unknown factors of special solutions determined by the fourth‐order ODE solvers, i.e., *a*_0_, *a_i_*, *b_i_* and λ in the general solution in Equation (3). Factors for the first‐ and second‐order Fourier series are saved separately. In the same manner, the mechanical inputs A_n_ and digital outputs Q_n_ are the data stored for the digital logic gates. Figure 5d showcases the mechanical systems in a patterned binary matrix. In the form of binary codes, these “0” and “1” units represent the unknow factors from the fourth‐order ODE solvers and the inputs and outputs from the digital logic gates, while “null” refers to the unused units as free space.

Here, we develop the computing‐storing system constituted of the logical operation, storage and interaction modules, which can perform the interaction between the logical operation and storage modules, namely storing the outputs of logical operation module in real time by the storage module, as demonstrated in Figure 5e. The interaction module can judge the conduction conditions of logical operation module and display the outputs through its green LED lights accordingly. Subsequently, the interaction module controls the extensive displacement of the linear actuator based on the outputs of logical operation module. The interaction module of the computing‐storing system is detailed in Note S8 (Supporting Information). When the logical operation module outputs Q = 0 (i.e., LED is off), the extensive displacement of the linear actuator is 0 mm and the storage module is in Φ_1_ that means “0” is stored. On the contrary, when the logical operation module outputs Q = 1 (i.e., LED is on), the extensive displacement of the linear actuator is 2 mm and the storage is Φ_3_ for storing “1”. Movie S5 (Supporting Information) takes the buffer and OR gates for examples to showcase the interaction between the logical operation and storage modules. In particular, when the mechanical input of buffer gate yields A = 0 (i.e., switch 1 is in Φ_1_), the LED is off (i.e., the logical operation module outputs Q = 0), and the linear actuator drives the storage module in Φ_1_ to store the output “0”. In contrast, when the buffer gate inputs A = 1 (i.e., switch 1 is in Φ_3_), the LED is on (i.e., the logical operation module outputs Q = 1) and the storage module is in Φ_3_ to store “1” (see Figure 5f). At present, the scientific computing‐storing system has yet to be developed due to the limitations of current technological competence. Specifically, storing the Fourier factors extracted via MATLAB in real time by the mechanical memories requires enormously complex control algorithm and device, and loading machine.

## 3 Discussion

This research developed the multi‐instably mechanical systems for the functions of scientific computing, logical operations and data storage. The mechanical fourth‐order ODE solvers obtained the special solutions for the fourth‐order ODEs by determining the unknow factors in the general solutions via comparing with the deformation shapes of multi‐instable beams subjected to bilateral constraints, and the results were validated with the existed theoretical solutions with satisfactory agreements. Subsequently, we converted the multi‐instable beams into the reconfigurable switches to build the digital logic gates of Buffer, NOT, AND, OR, NAND, NOR, XOR, and XNOR. To obtain the stable mechanical memories by the multi‐instable response, we embedded the beams in the fixed frames to realize the storage units with tailorable deformation shapes (i.e., Φ_1_ and Φ_3_). The units were assembled into *m×n* storage matrix to store the ASCII characters and arbitrary integral numbers. Eventually, the mechanical systems with integrated functions were demonstrated by combining the computation module of the fourth‐order ODE solvers and digital logic gates with the storage module of the mechanical memories. At the current stage, the reported computing‐storing system can accomplish the interaction between the logical operation and storage modules.

This study innovatively exploited the physical substrate of multi‐instability to realize the mechanical systems with the functions of scientific computing, logical operations and data storage. Notably, the proposed fourth‐order ODE solvers significantly broaden the field as mechanical computing is still in its infancy for scientific computing such as ODE solving, let alone incorporating it into the integrated system with the other mechanical functions of logical operations and data storage. Although the prototypes in this study are still at the early stage, the reported multi‐instably mechanical systems are expected to lead the development direction of mechanical devices with multifunctionality, integration and well environmental perception.

## 4 Experimental Section

### Theoretical Analysis of Slender Beams Subjected to Bilateral Constraints

Bilaterally confined elastic beams under axial compression can achieve higher buckling modes beyond the unrestrained elastic beams. The multi‐instability of a bi‐walled elastic beam with the length, width and thickness of *L*, *W* and *t* under axial compression includes three phases (Figure 1a). In phase 1, the beam shortens axially under axial pressure *F*. In phase 2, as *F* reaches the critical buckling force, the beam buckles and deflects laterally. In phase 3, the maximum transverse deflection is limited to *h*_0_ − *t* (*h*_0_ is the gap between two constraints) under bilateral constraints, which results in the transition from first buckling mode (Φ_1_) to higher buckling modes. The multi‐instability of bilaterally confined elastic beams is governed by^[^ ^37^ ^]^

(4) $$\frac{d^{4}w\left(x\right)}{dx^{4}}+\frac{F}{EI}\frac{d^{2}w\left(x\right)}{dx^{2}}=0$$

where *w*(*x*) denotes the transverse deflection of elastic beam at *x* (0 ≤ *x* ≤ *L*) from the loading end, *E* and $I=\frac{Wt^{3}}{12}\;$ present the Young's modulus and moment of inertia of elastic beam. Equation (4) satisfies the boundary conditions as

(5)

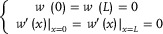




The closed‐form analytical solutions of Equation (4) are obtained by the mode superposition method as

(6) $$\begin{array}{ccc} w\left(x\right) & = & \sum_{j=1,3,5,\text{…}}^{\infty }A_{j}h\left[1-\cos\left(\frac{N_{j}x}{L}\right)\right]\\ & & +\sum_{j=2,4,6,\text{…}}^{\infty }A_{j}h\left[1-\frac{2x}{L}-\cos\left(\frac{N_{j}x}{L}\right)+\frac{2}{N_{j}}\sin\left(\frac{N_{j}x}{L}\right)\right] \end{array}$$

The detailed theoretical deduction, and determination of coefficients *A_j_*, *N_j_* (j=1,2,3,…) are provided in Note S2 (Supporting Information).

### Fabrication of the Bilaterally Constrained Beams made of PLA and PLA‐CB by 3D Printing

The components of reported fourth‐order ODE solvers, digital logic gates and mechanical memories are fabricated through 3D printing via a dual extruder 3D printer (i.e., Rasie3D Pro2). In particular, the PLA is selected to fabricate the nonconductive and white components, while the PLA‐CB is chosen to prepare the conductive and black components. The microstructures of PLA and PLA‐CB filaments captured by a scanning electron microscope (SEM) are presented in Note S1 (Supporting Information). The platform temperature, printing temperature and filling speed are separately set as 60 °C, 205 °C and 60 mm s^−1^ during the printing. The filling rate is set as 60% for the elastic beams, while the filling rate is set as 50% for the rest of components. For the single‐unit and two‐units ODE solvers, and logic switches 1 and 2, the printed components are glued using the instant‐drying adhesive to construct the functional devices. For the storage units and storage matrix, the printed components are simply spliced to form the functional devices to meet the requirements of detachability, reconfigurability and efficient storage.

### Mechanical Testing for Stable Deformation Shapes in Φ_1_ and Φ_3_ with Well Controllability

A computerized electronic fatigue testing machine manufactured by Han Shen Automation (Jinan) Co., Ltd was used to apply the compressive load in the displacement‐control mode, and the loading rate is set as 5 mm min^−1^. Two 3D printed PLA rigid fixtures are connected to the testing machine to ensure the uniform distribution and stability of displacement load. For the logic switches, the displacement and force intervals that form the buckling modes of Φ_1_ and Φ_3_ are determined by the obtained force‐displacement relationships (see Note S4, Supporting Information). In order to control the geometric design of storage unit that ensure the Φ_1_ and Φ_3_ happen, the bi‐walled PLA‐CB beam with the identical structural parameters as the storage unit is tested to access the corresponding displacement intervals of Φ_1_ and Φ_3_ (see Note S7, Supporting Information).

### Deformation Shape Extraction and Comparison

For the purpose of accurately capturing the output solutions given by the fourth‐order ODE solvers (i.e., the configurations of deformed beams), the deformation shape extraction is conducted using the MATLAB software. First, recording the final deformed configurations of fourth‐order ODE solvers under the given input parameters by the camera. Subsequently, applying the functions of imread, imshow and ginput in MATLAB to read the image, display the image and capture the coordinates of specified points on the deformed beams, respectively. Finally, determining the factors *a*_0_, *a_i_*, *b_i_* and λ of fitted Fourier series by comparing with the obtained coordinates using the MATLAB's curve fitting toolbox (i.e., cftool). The expressions of output solutions of all test cases are summarized in Note S3 (Supporting Information).

## Conflict of Interest

The authors declare no conflict of interest.

## Author Contributions

P.J. designed and supervised the research. P.J. and J.W. contributed to the conception and established the theoretical models. J.W. and C.Z. conducted the structural design, carried out the experiments, and analyzed the source data. Q.Z. supervised the research. J.W. and P.J. wrote the initial manuscript. J.W. and Q.Z. revised the manuscript with contributions from all authors

## Supporting information



Supporting Information

Supplemental Movie 1

Supplemental Movie 2

Supplemental Movie 3

Supplemental Movie 4

Supplemental Movie 5

## Data Availability

The data that support the findings of this study are available in the Supplementary Information of this article.
