# Supplementary material for: Multi‐Instably Mechanical Systems for Computing‐Storing Functions
Source: Adv Sci (Weinh). 2025 Sep 25;13(2):e10880. doi: 10.1002/advs.202510880 (PMC12786300; doi:10.1002/advs.202510880)
Supplement: Supplementary file 1 — Supporting Information [file ADVS-13-e10880-s004.docx]

**Supplementary Information for**

**Multi-instably Mechanical Systems with Computing-Storing Functions**

Jiajun Wang, Chenjie Zhang, Qianyun Zhang, Pengcheng Jiao

Correspondence to: [qianyunz@nmsu.edu](mailto:qianyunz@nmsu.edu) (Q. Zhang) and [pjiao@zju.edu.cn](mailto:pjiao@zju.edu.cn) (P. Jiao)

# Supplementary Note 1: Microstructures of PLA-CB and PLA filaments

The microstructures of 3D printing filaments PLA-CB and PLA are obtained and compared by a scanning electron microscope (SEM), as presented in Fig. S1. It can be seen that the micromorphology of PLA-CB filament is rough and porous, while the micromorphology of PLA filament is smooth and dense. The conductibility of PLA-CB originates from the mixed filler of conductive carbon black (Fig. S1a).


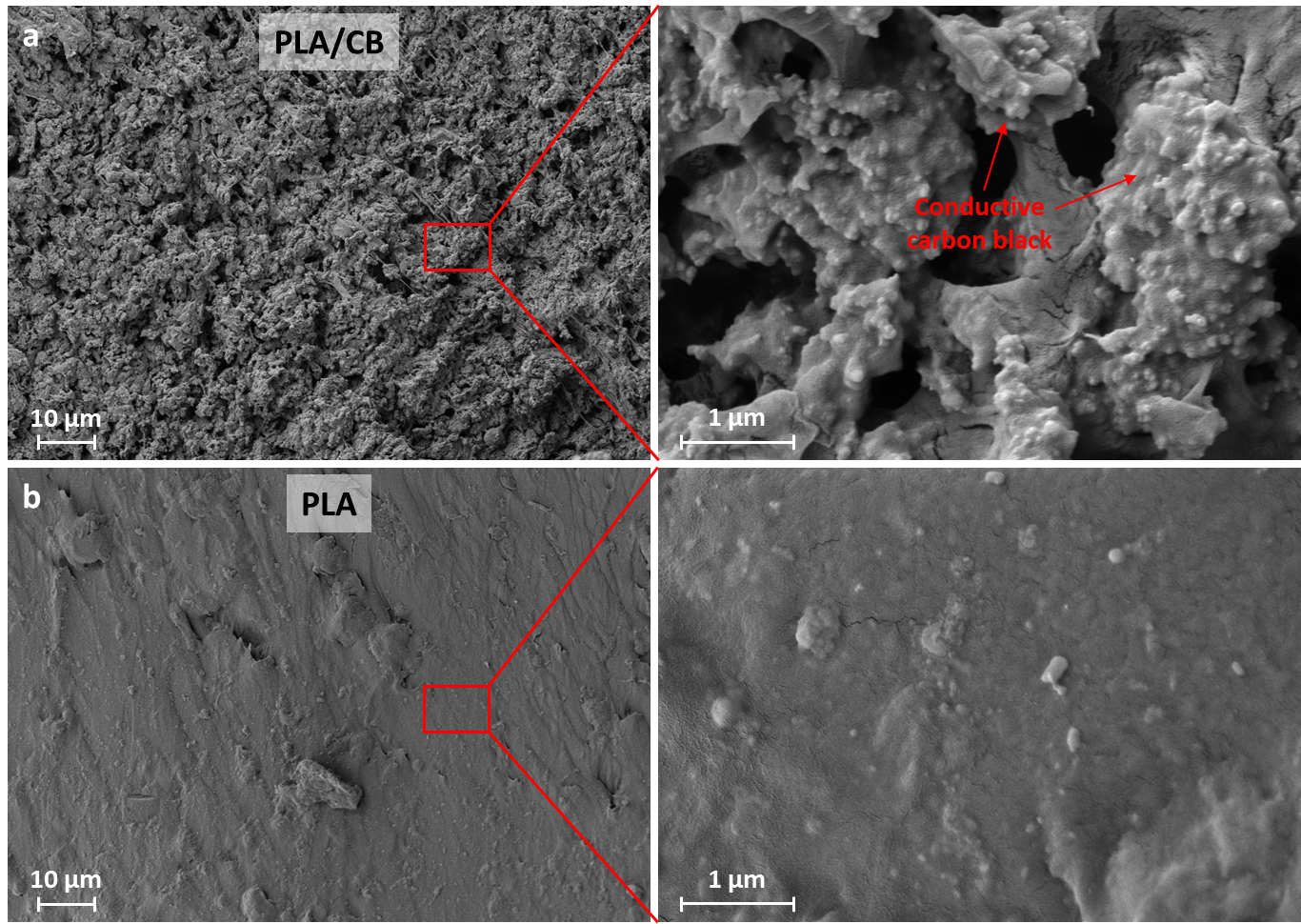


**Fig. S1. Microstructures of 3D printing filaments PLA-CB and PLA. a** PLA-CB filament. **b** PLA filament.

# Supplementary Note 2: Characterizations of multi-instability of bi-walled elastic beam

Fig. S2a presents the experimental characterization of multi-instability of bilaterally confined PLA beam with the structural parameters of $L=103 mm$, $W=8 mm$, $t=0.5 mm$ and $h_{0}=5 \mathrm{mm}$. The experimental phenomenon shows that after the beam reaches the maximum transverse deflection and enters the first buckling mode ($\Phi_{1}$), the contact area between beam and constraint gradually expands as the axial pressure increases, and then the beam exhibits the fatten $\Phi_{1}$. However, the relatively stable state $\Phi_{1}$ cannot be maintained due to the continuous input of mechanical energy from the axial pressure, so that the beam transforms into the third buckling mode ($\Phi_{3}$). The mechanical responses (i.e., force-displacement and energy-displacement relationships) of bi-walled PLA beam with the structural parameters of $L=105 mm$, $W=8 mm$, $t=0.5 mm$ and $h_{0}=2 \mathrm{mm}$ are demonstrated in Fig. S2b. It can be seen that the axial pressure significantly decreases during the buckling mode transition, which leads to the growth slope down of potential energy.


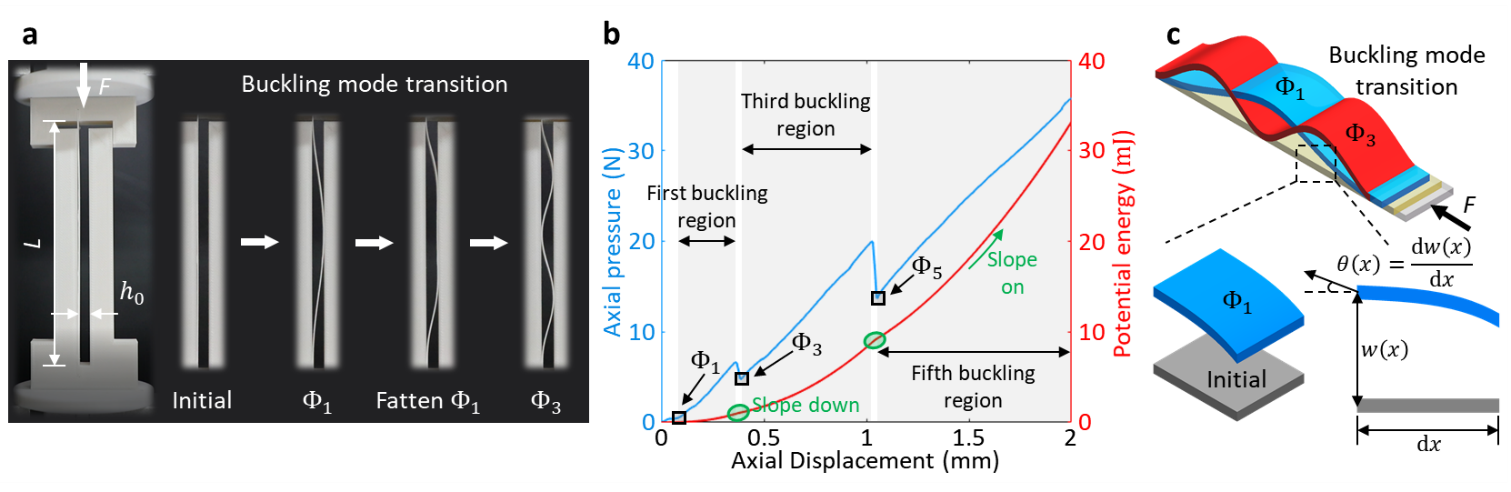


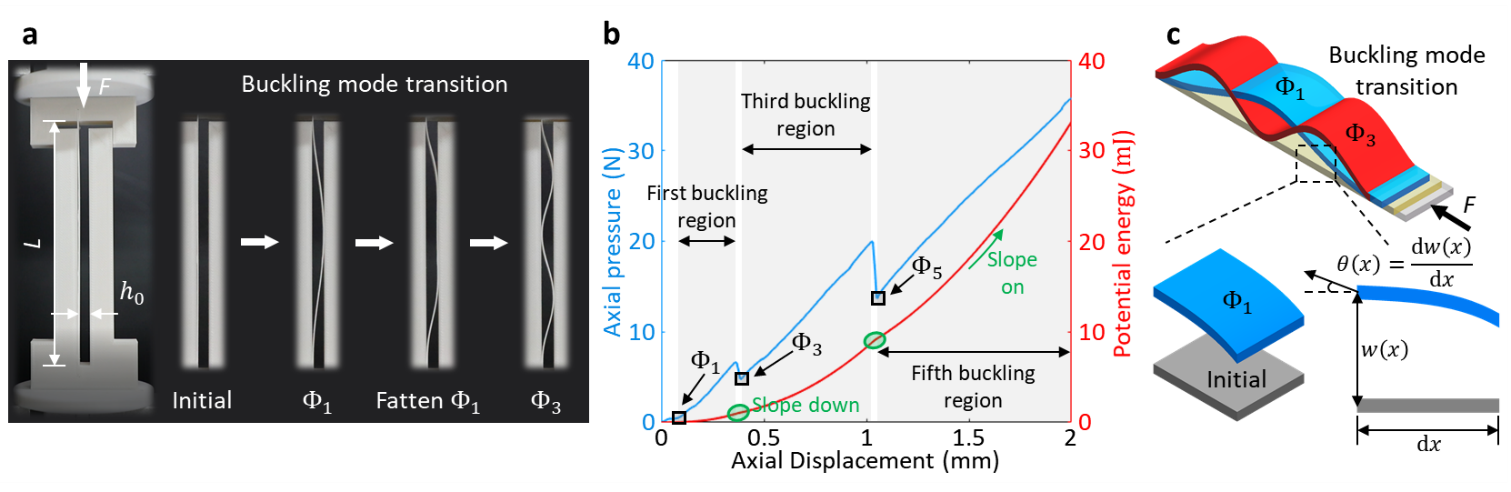


**Fig. S2. Characterizations of multi-instability of bilaterally confined elastic beams. a** Experimental characterization. **b** Mechanical responses. **c** Theoretical foundation.

Next, we theoretically investigate the multi-instability of bilaterally confined elastic beams and obtain the closed-form analytical solutions. Fig. S2c explains the theoretical foundation. The multi-instability of fully elastic and recoverable bi-walled beams is governed by (*1*-*2*)

| $\frac{d^{4}W\left( X \right)}{dX^{4}}+N^{2}\frac{d^{2}W\left( X \right)}{dX^{2}}=0.$ | (S1) |
| --- | --- |

Note that Eq. (S1) is normalized, where the coefficient $N^{2}=\frac{FL^{2}}{EI}-\frac{wth^{2}}{2I}\int_{0}^{1} \left( \frac{dW\left( X \right)}{dX} \right)^{2}dX$, and normalized physical quantities $X=\frac{x}{L}$ and $W\left( X \right)=\frac{w\left( x \right)}{h}$. The boundary conditions of Eq. (S1) are

| $\left\{ \begin{aligned} W\left( 0 \right)=W\left( 1 \right)=0 \\ \left. \frac{dW\left( X \right)}{dX} \right\vert_{X=0}=\left. \frac{dW\left( X \right)}{dX} \right\vert_{X=1}=0 \end{aligned} \right..$ | (S2) |
| --- | --- |

Jiao et al. used the mode superposition method to characterize the normalized transverse deflection as (*3*)

| $W\left( X \right)=\sum_{j=1}^{\infty} A_{j}\psi_{j}\left( X \right),$ | (S3) |
| --- | --- |

where $\psi_{j}\left( X \right)$ denotes the buckling mode function, and $A_{j}$ presents the coefficient that describes the contribution of buckling mode function to the normalized transverse deflection. There are two categories of buckling mode functions, i.e., symmetric and antisymmetric. The symmetric buckling mode functions are

| $\left\{ \begin{aligned} \psi_{j}\left( X \right)=1-\cos\left( N_{j}X \right) \\ N_{j}=\left( j+1 \right)\pi\end{aligned} \right., j=1, 3, 5,\ldots$ | (S4) |
| --- | --- |

while the antisymmetric buckling mode functions are

| $\left\{ \begin{aligned} \psi_{j}\left( X \right)=1-2X-\cos\left( N_{j}X \right)+\frac{2\sin\left( N_{j}X \right)}{N_{j}} \\ N_{j}=2.86\pi, 4.92\pi, 6.94\pi, 8.95\pi, \ldots\end{aligned} \right., j=2, 4, 6, \ldots$ | (S5) |
| --- | --- |

Substituting Eqs. (S4) and (S5) into Eq. (S3) leads to

| $W\left( X \right)=\sum_{j=1, 3, 5, \ldots}^{\infty} A_{j}\left[ 1-\cos\left( N_{j}X \right) \right]+\sum_{j=2, 4, 6, \ldots}^{\infty} A_{j}\left[ 1-2X-\cos\left( N_{j}X \right)+\frac{2\sin\left( N_{j}X \right)}{N_{j}} \right].$ | (S6) |
| --- | --- |

Therefore, the actual transverse deflection can be written as

| $w\left( x \right)=\sum_{j=1, 3, 5, \ldots}^{\infty} A_{j}h\left[ 1-\cos\left( \frac{N_{j}x}{L} \right) \right]+\sum_{j=2, 4, 6, \ldots}^{\infty} A_{j}h\left[ 1-\frac{2x}{L}-\cos\left( \frac{N_{j}x}{L} \right)+\frac{2}{N_{j}}\sin\left( \frac{N_{j}x}{L} \right) \right].$ | (S7) |
| --- | --- |

Eq. (S7) are the closed-form analytical solutions that to be compared with the output solutions from DE solvers. In order to determine the unknown coefficients $A_{j}$ ($j=1, 2, 3, \ldots$), the energy minimization method that minimizes the total potential energy of bilaterally confined elastic beams is applied. The normalized total potential energy $\Omega$ includes the bending strain energy $U_{b}$, compressive strain energy $U_{c}$ and potential energy from external axial pressure $U_{e}$, which can be expressed as

| $\left\{ \begin{aligned} U_{b}=\frac{1}{2}\int_{0}^{1} \left( \frac{d^{2}W\left( X \right)}{dX^{2}} \right)^{2}dX \\ U_{c}=\frac{PD_{c}}{2} \\ U_{e}=-\frac{PD}{2} \end{aligned} \right..$ | (S8) |
| --- | --- |

The normalized physical quantities in Eq. (S8) are given as

| $\left\{ \begin{aligned} P=\frac{FL^{2}}{EI} \\ D_{c}=\frac{\Delta_{c}L}{h^{2}}=\frac{FL^{2}}{Ewth^{2}} \\ D=\frac{\Delta L}{h^{2}}=D_{c}+\frac{1}{2}\int_{0}^{1} \left( \frac{dW\left( X \right)}{dX} \right)^{2}dX=\frac{FL^{2}}{Ewth^{2}}+\frac{1}{2}\int_{0}^{1} \left( \frac{dW\left( X \right)}{dX} \right)^{2}dX \end{aligned} \right..$ | (S9) |
| --- | --- |

Substituting Eq. (S9) into Eq. (S8), the normalized total potential energy is obtained as

| $\Omega=U_{b}+U_{c}+U_{e}=\frac{1}{2}\int_{0}^{1} \left( \frac{d^{2}W\left( X \right)}{dX^{2}} \right)^{2}dX-\frac{FL^{2}}{2EI}\int_{0}^{1} \left( \frac{dW\left( X \right)}{dX} \right)^{2}dX.$ | (S10) |
| --- | --- |

Taking Eq. (S6) into Eq. (S10) yields

| $\Omega=\frac{1}{2}\int_{0}^{1} \left\{ \sum_{j=1, 3, 5, \ldots}^{\infty} A_{j}{N_{j}}^{2}\cos\left( N_{j}X \right)+\sum_{j=2, 4, 6, \ldots}^{\infty} A_{j}\left[ {N_{j}}^{2}\cos\left( N_{j}X \right)-2N_{j}\sin\left( N_{j}X \right) \right] \right\}^{2}dX-\frac{FL^{2}}{2EI}\int_{0}^{1} \left\{ \sum_{j=1, 3, 5, \ldots}^{\infty} A_{j}N_{j}\sin\left( N_{j}X \right)+\sum_{j=2, 4, 6, \ldots}^{\infty} A_{j}\left[ N_{j}\sin\left( N_{j}X \right)+2\cos\left( N_{j}X \right)-2 \right] \right\}^{2}dX.$ | (S11) |
| --- | --- |

The coefficients $A_{j}$ ($j=1, 2, 3, \ldots$) can be calculated by

| $\left\{ \begin{aligned} \mathrm{Min}\left[ \Omega\left( A_{j} \right) \right], j=1, 2, 3, \ldots\\ 0\leq W\left( X \right)\leq1 \end{aligned} \right..$ | (S12) |
| --- | --- |

The Nelder-Mead algorithm is utilized to numerically solve $A_{j} \left( j=1, 2, 3, \ldots\right)$ in the energy minimization problem of Eq. (S12). Substituting the results of $A_{j} \left( j=1, 2, 3, \ldots\right)$ into Eqs. (S6) and (S7), $W\left( X \right)$ and $w\left( x \right)$ are determined.

# Supplementary Note 3: Output solutions from fourth-order ODE solvers


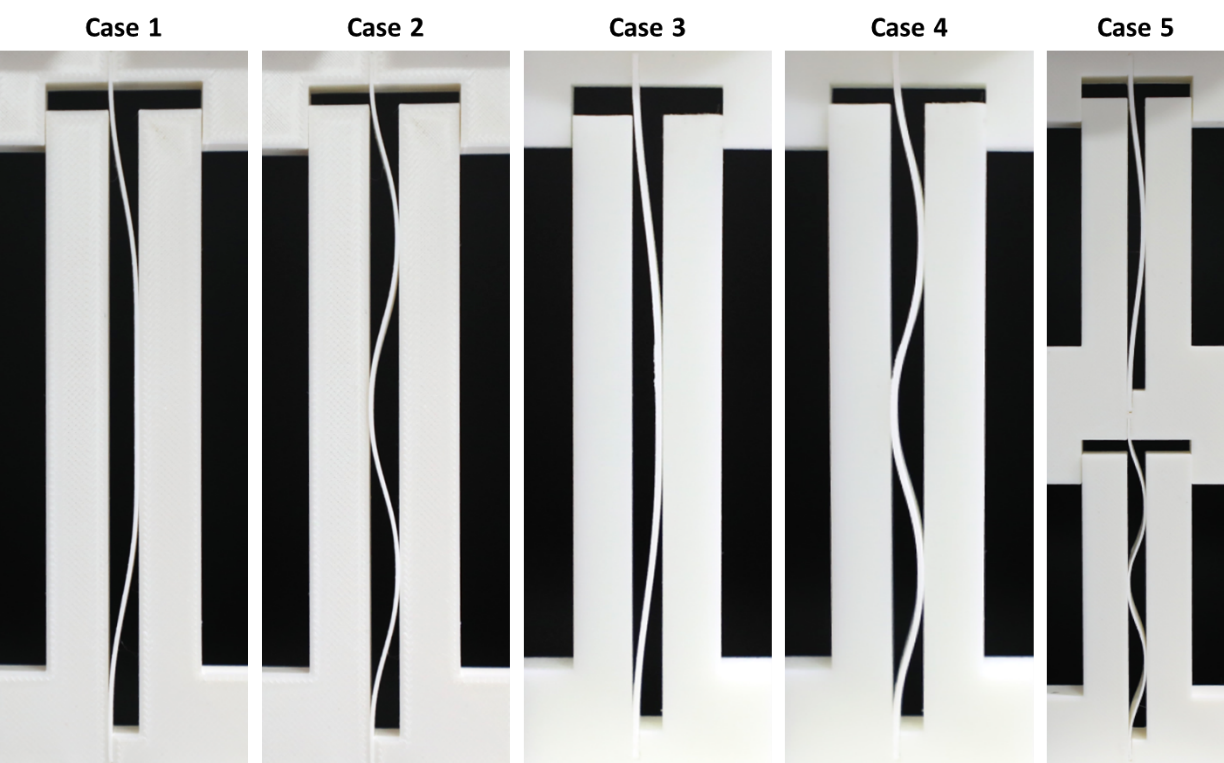


**Fig. S3. Output solutions from fourth-order ODE solvers.**

Fig. S3 presents the buckling configurations of fourth-order ODE solvers during the solving of five test cases. For cases 1 and 2, the structural parameters of bi-walled PLA beam are $L=105 \mathrm{mm}$, $W=8 \mathrm{mm}$, $t=0.5 \mathrm{mm}$ and $h=4.5 \mathrm{mm}$, and the material parameter (i.e., Young’s modulus) is determined as $E=1218.33 \mathrm{MPa}$ by the tensile tests. The subjected axial pressure $F$ and quantity of collected coordinates $N$ are set as 4.5 N, 20 for case 1, and 8 N, 40 for case 2, respectively. For cases 3 and 4, the structural and material parameters of bi-walled PLA beam are $L=105 \mathrm{mm}$, $W=8 \mathrm{mm}$, $t=1 \mathrm{mm}$, $h=4 \mathrm{mm}$ and $E=876.92 \mathrm{MPa}$. $F$ and $N$ are set as 6.2 N, 20 for case 3, and 39.4 N, 40 for case 4. For case 5, the PLA beam in the first layer possesses the parameters of $L=70 \mathrm{mm}$, $W=8 \mathrm{mm}$, $t=1 \mathrm{mm}$, $h=3 \mathrm{mm}$, $E=879.55 \mathrm{MPa}$ and $N=20$, while the parameters of PLA beam in the second layer are $L=70 \mathrm{mm}$, $W=8 \mathrm{mm}$, $t=0.5 \mathrm{mm}$, $h=3 \mathrm{mm}$, $E=999.45 \mathrm{MPa}$ and $N=30$. $F$ is set as 12.1 N. The output solutions given by fourth-order ODE solvers are determined by collecting the coordinates on the buckled beam and fitting using the MATLAB software, which are summarized in Table S1.

**Table S1. Output solutions from fourth-order ODE solvers of five test cases.**

| **Cases** | | **Types of Fourier series** | **Expressions** |
| --- | --- | --- | --- |
| 1 | | 2^nd^ order | $2.919\times{10}^{-3}-2.265\times{10}^{-3}\cos\left( 59.84x \right)+1.771\times{10}^{-4}\sin\left( 59.84x \right)-6.273\times{10}^{-4}\cos\left( 119.68x \right)+1.363\times{10}^{-4}\sin\left( 119.68x \right)$ |
| 2 | | 1^st^ order | $2.287\times{10}^{-3}-2.274\times{10}^{-3}\cos\left( 119.7x \right)+1.47\times{10}^{-4}\sin\left( 119.7x \right)$ |
| 3 | | 2^nd^ order | $2.306\times{10}^{-3}-1.984\times{10}^{-3}\cos\left( 59.84x \right)+4.308\times{10}^{-5}\sin\left( 59.84x \right)-3.157\times{10}^{-4}\cos\left( 119.68x \right)+2.241\times{10}^{-5}\sin\left( 119.68x \right)$ |
| 4 | | 1^st^ order | $2.084\times{10}^{-3}-1.909\times{10}^{-3}\cos\left( 119.7x \right)+1.031\times{10}^{-4}\sin\left( 119.7x \right)$ |
| 5 | First layer | 1^st^ order | $1.485\times{10}^{-3}-1.409\times{10}^{-3}\cos\left( 89.76x \right)+2.161\times{10}^{-4}\sin\left( 89.76x \right)$ |
|  | Second layer | 1^st^ order | $1.797\times{10}^{-3}-1.76\times{10}^{-3}\cos\left( 179.5x \right)+1.154\times{10}^{-4}\sin\left( 179.5x \right)$ |

# Supplementary Note 4: Compressive performance of composite switches

Fig. S4 presents the compressive performance (i.e., force-displacement relationships) of switches 1 (Fig. S4b) and 2 (Fig. S4c). For Switch 1, when in the first buckling region, the conductive beam contacts with the nonconductive component, which causes the switch to open. Yet, when in the third buckling region, the conductive beam connects the conductive components 1 and 2, so that the switch is in the closed state. In contrast, for Switch 2, the conductive beam connects the conductive components 1 and 2 (i.e., the switch is closed) when in the first buckling region, while the conductive beam contacts with the nonconductive component (i.e., the switch is open) when in the third buckling region.


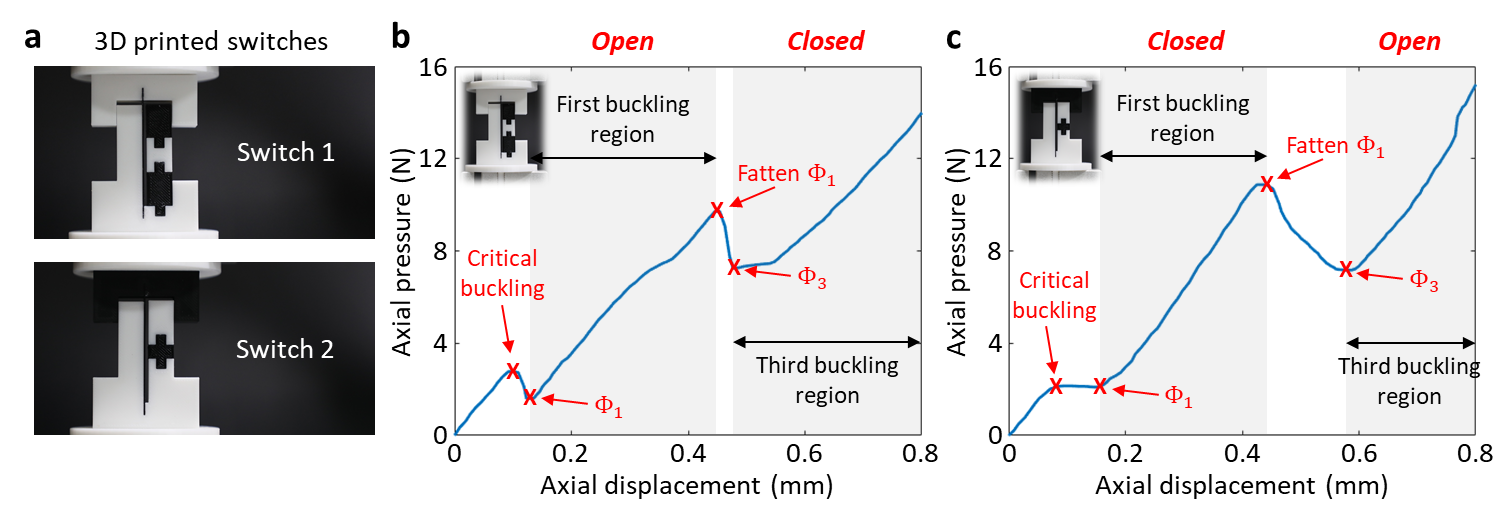

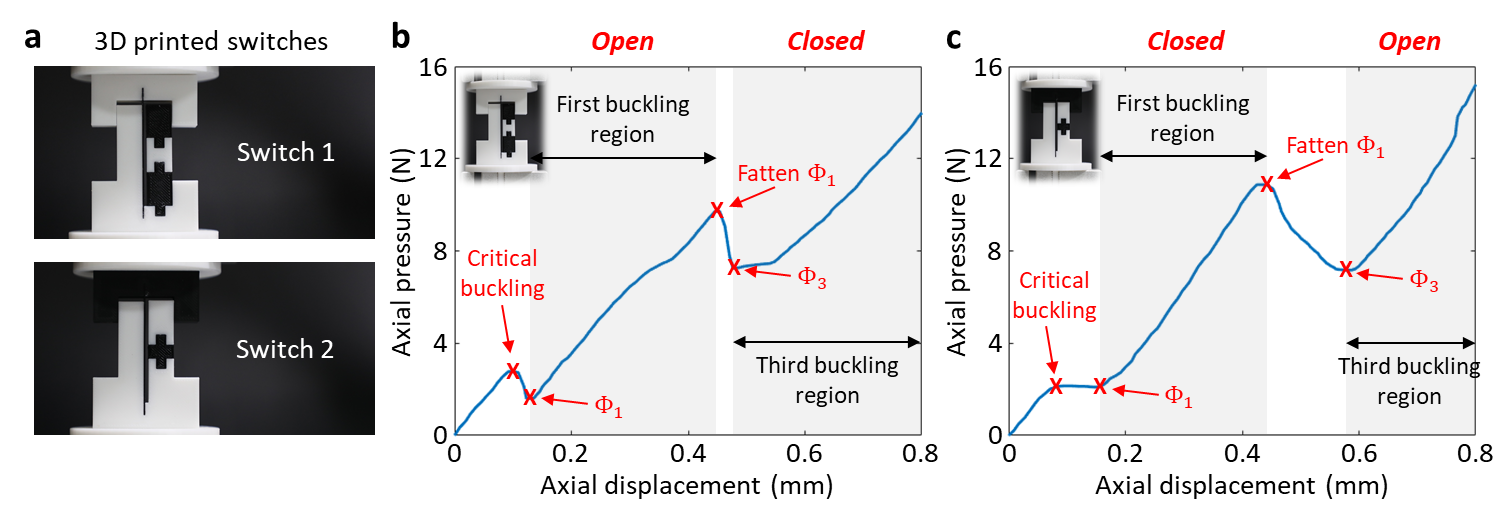


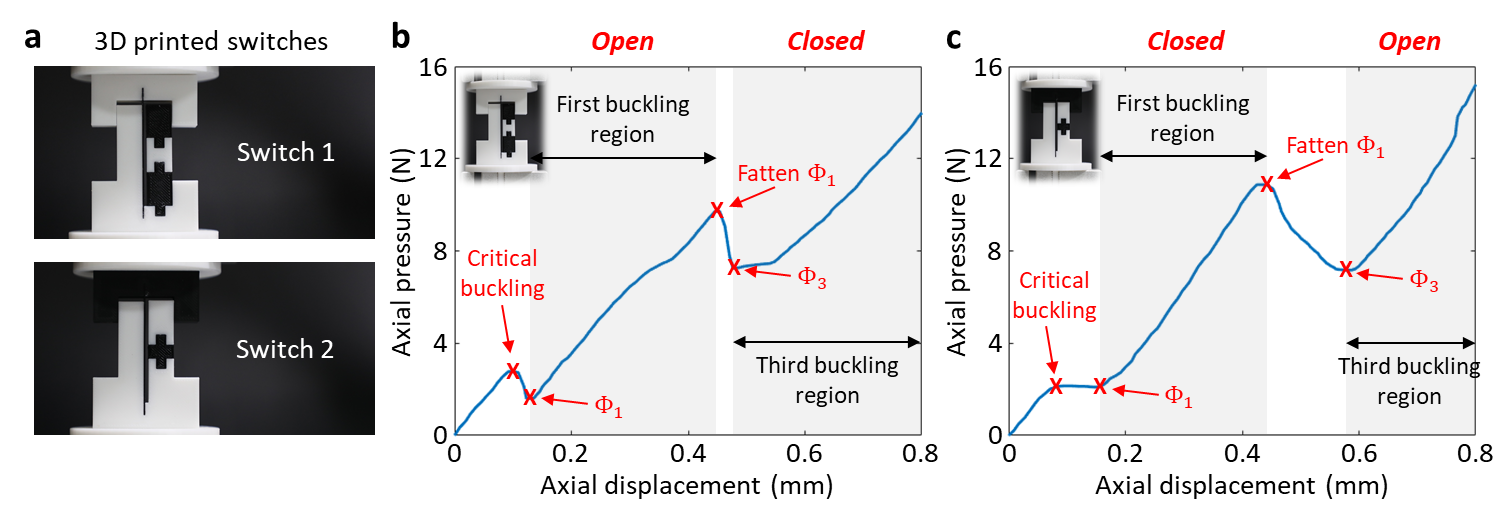


**Fig. S4. Compressive tests of composite switches. a** 3D printed composite switches. Compressive performance of (**b**) switches 1 and (**c**) 2.

# Supplementary Note 5: Circuit diagrams of OR and NOR gates

Figs. S5 presents the circuit diagrams of the OR and NOR gates that are not included in the main text.


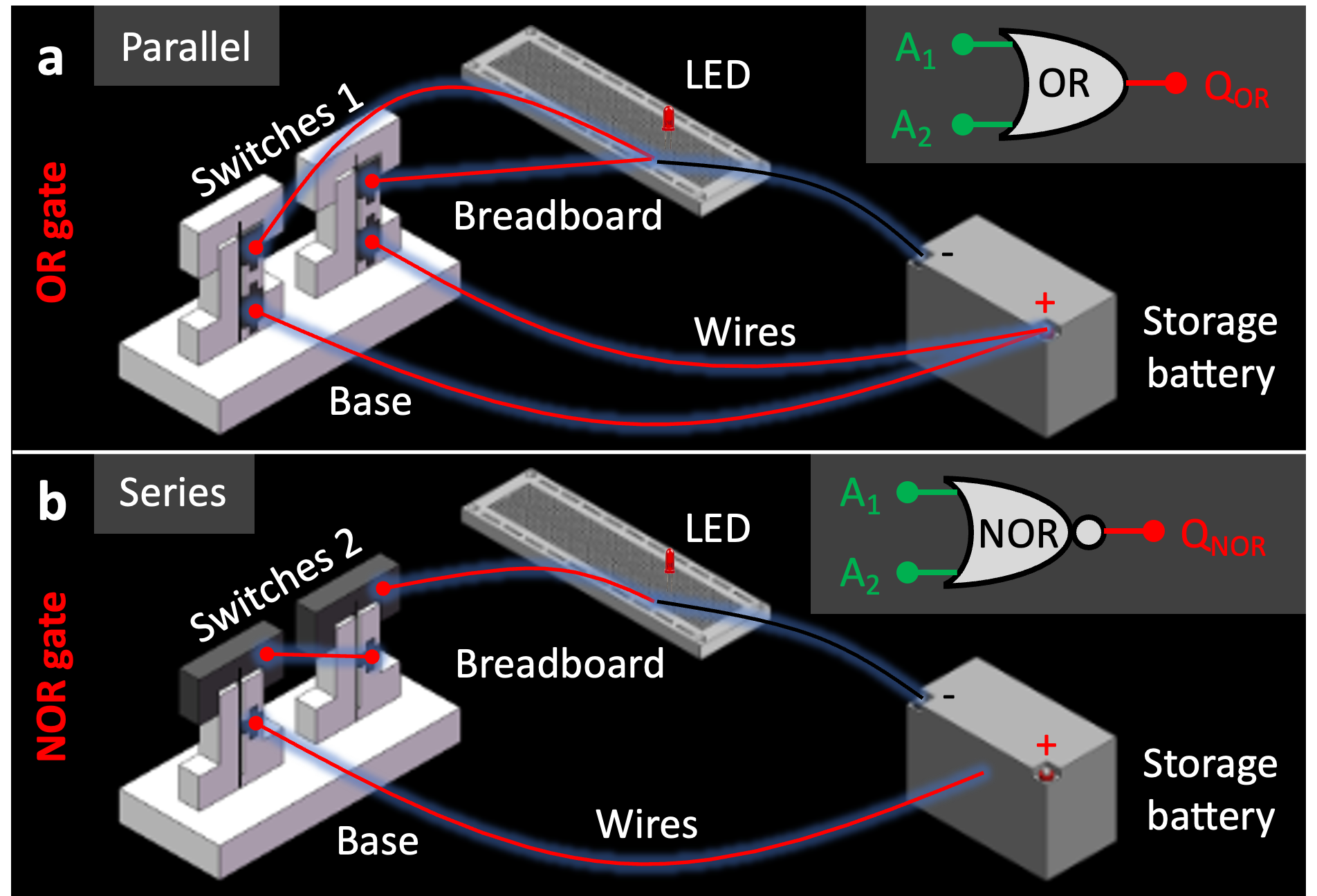


**Fig. S5. Circuit diagrams. a** OR gate. **b** NOR gate.

# Supplementary Note 6: Experimental demonstrations of basic digital logic gates and half adder

Figs. S6 and S7 distinctly present the experimental demonstrations of digital logic gates with single mechanical input and double mechanical inputs, respectively. Fig. S8 distinctly shows the experimental demonstrations of the half adder.


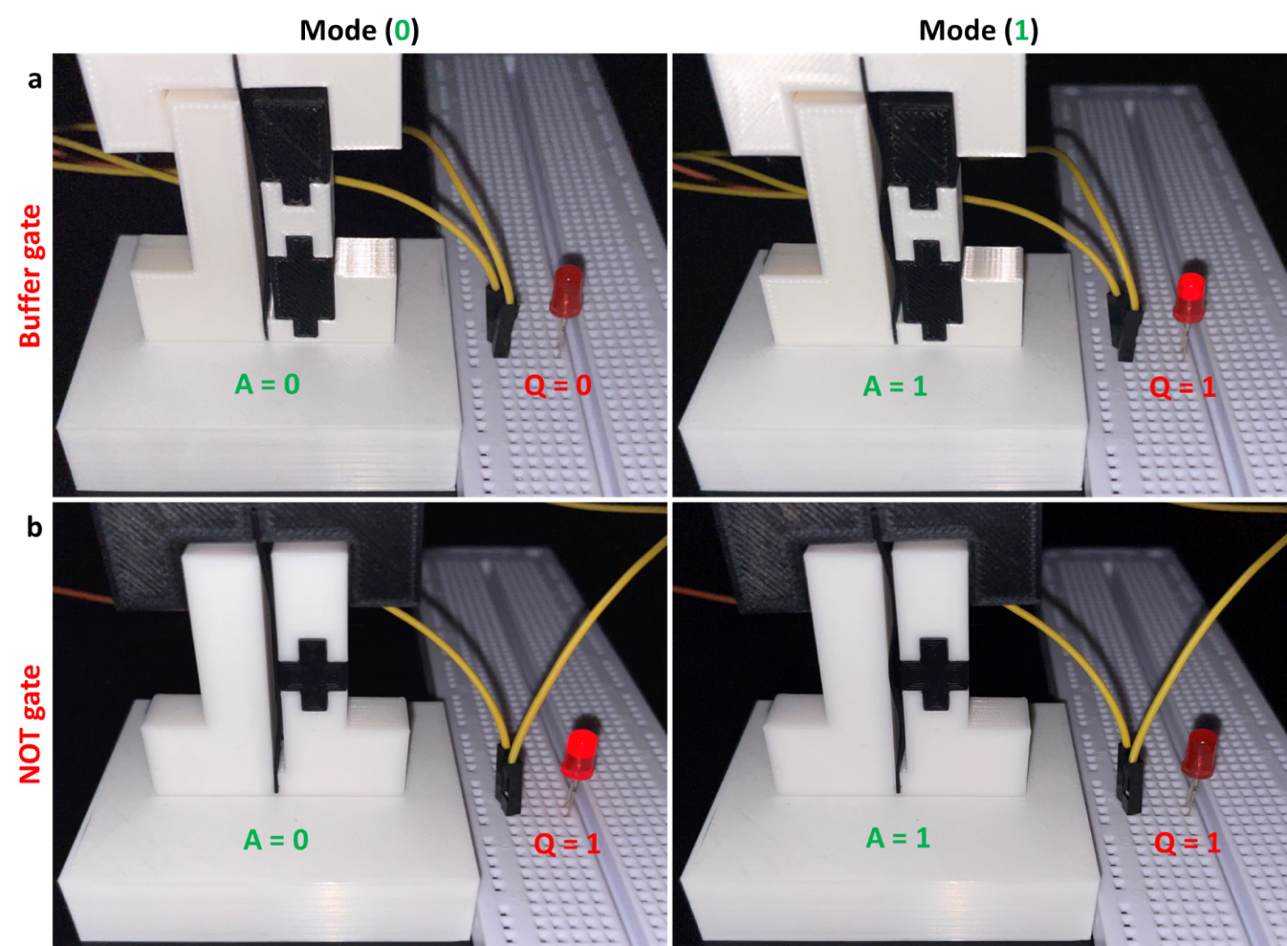


**Fig. S6. Experimental demonstrations of digital logic gates with single mechanical input. a** Buffer gate. **b** NOT gate.


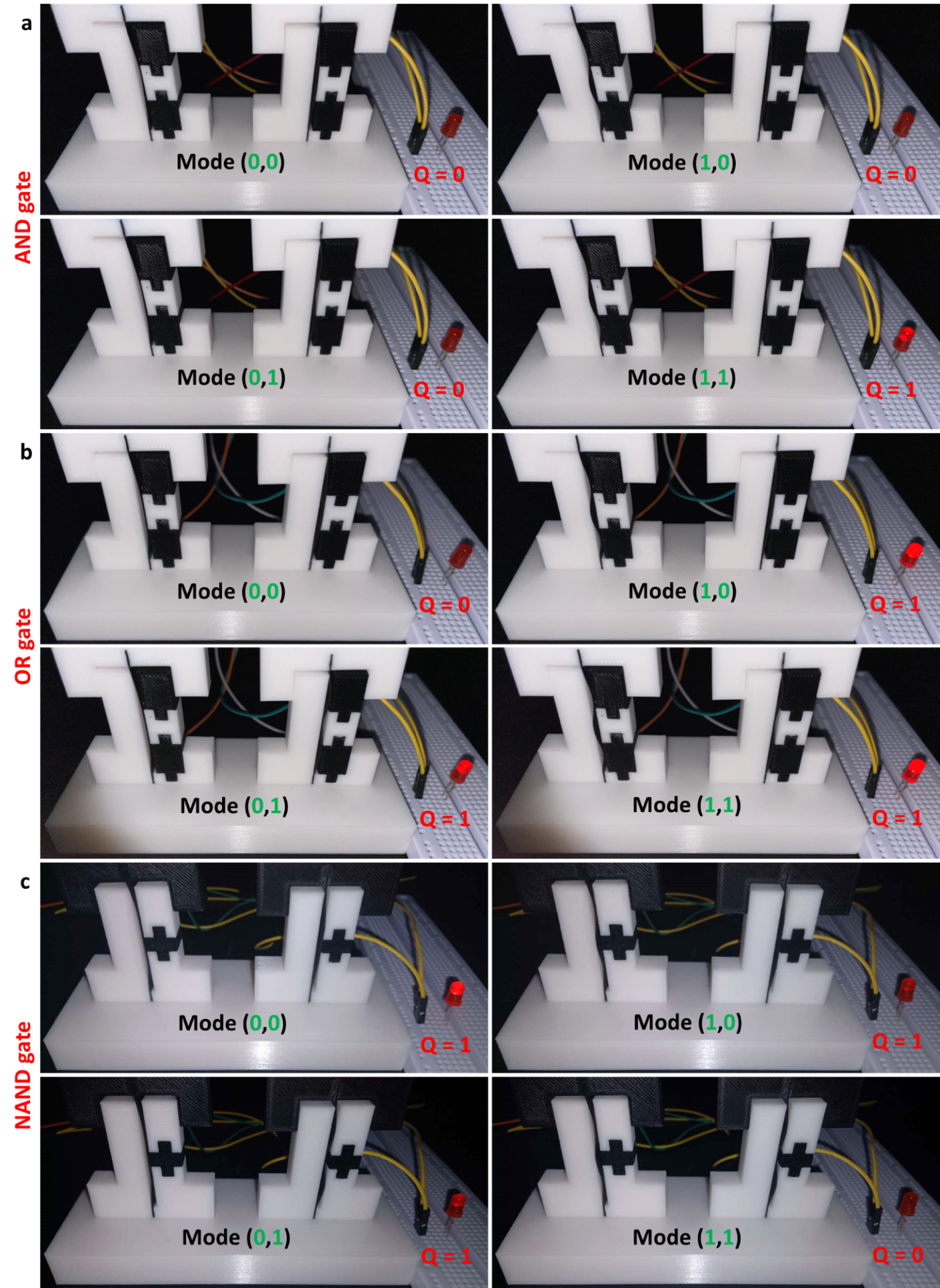


**Fig. S7. Experimental demonstrations of digital logic gates with double mechanical input. a** AND gate. **b** OR gate. **c** NAND gate. **d** NOR gate. **e** XOR gate. **f** XNOR gate.


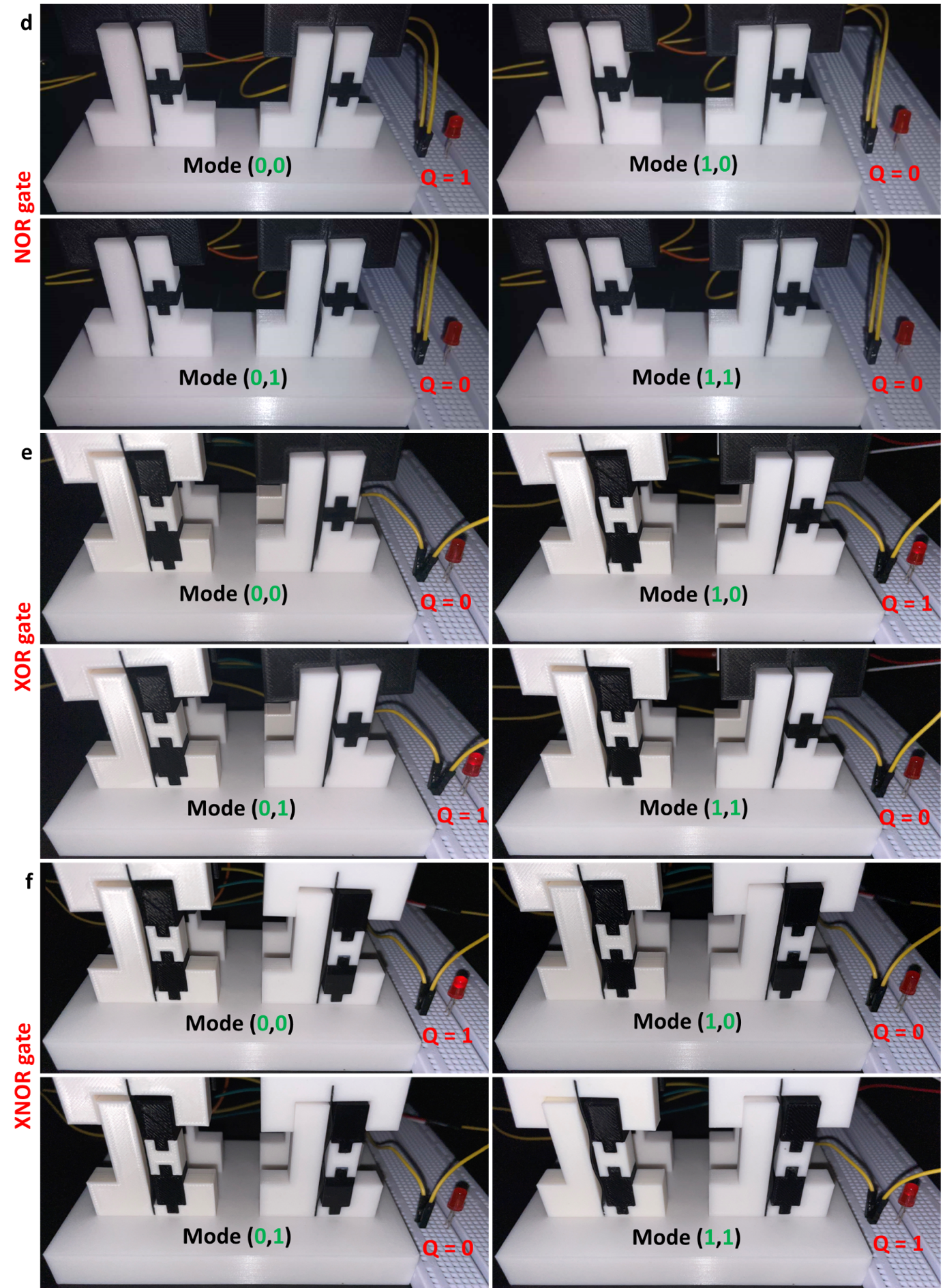


**Fig. S7.** (*Continued*).


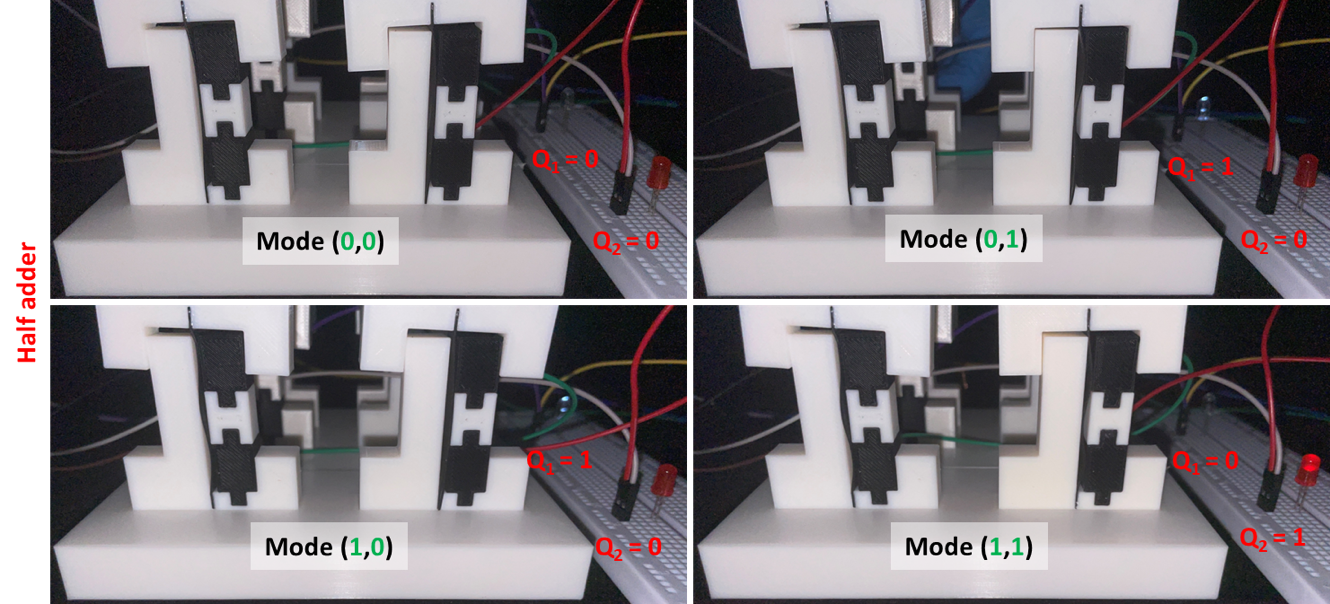


**Fig. S8. Experimental demonstrations of the half adder.**

# Supplementary Note 7: Determining the head height for different buckling modes by compression test

Fig. S9 presents the force-displacement relationship of bilaterally confined PLA-CB beam with the identical structural parameters (i.e., $L=30 mm$, $W=5 mm$, $t=0.5 mm$ and $h_{0}=2 \mathrm{mm}$) as storage unit, which determines the first and third buckling regions. And thus, 3.8 mm (i.e., the axial displacement = 0.3 mm) and 4.3 mm (i.e., the axial displacement = 0.8 mm) are selected as the designs of head height to achieve the buckling modes of $\Phi_{1}$ and $\Phi_{3}$, respectively. Table S2 summarizes the geometric parameters for configuring different buckling modes of storage unit.


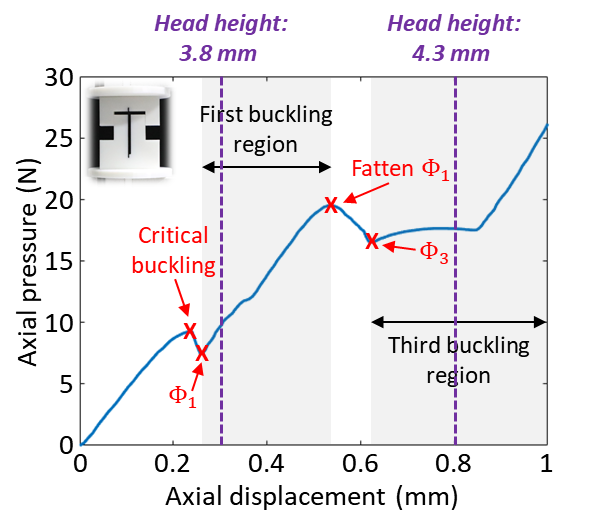


**Fig. S9. Compressive test of bi-walled PLA-CB beam with the identical structural parameters of storage unit.**

**Table S2. Geometric parameters for different buckling modes.**

| **Buckling mode** | $\boldsymbol{L}$ **(mm)** | $\boldsymbol{W}$ **(mm)** | $\boldsymbol{t}$ **(mm)** | $\boldsymbol{h}_{\boldsymbol{0}}$ **(mm)** | **Head height (mm)** | **Loading displacement (mm)** |
| --- | --- | --- | --- | --- | --- | --- |
| $\Phi_{1}$ | 30 | 5 | 0.5 | 2 | 3.8 | 0.3 |
| $\Phi_{3}$ |  |  |  |  | 4.3 | 0.8 |

# Supplementary Note 8: Construction of interaction module for logic-storing integrated system

The STM32F103C8T6 core board and step motor driver board were used to construct the interaction module for the logic-storing integrated system, as presented in Fig. S10. In particular, the core board can judge the conduction conditions of mechanical input module of logical operation module, while displaying the output results of logical operation module by its green LED light. Subsequently, the core board sends instructions to the step motor driver board to drive the linear actuator to extend or retract, and then the storage module deforms to the corresponding buckling modes to store the outputs of logical operation module in real time.


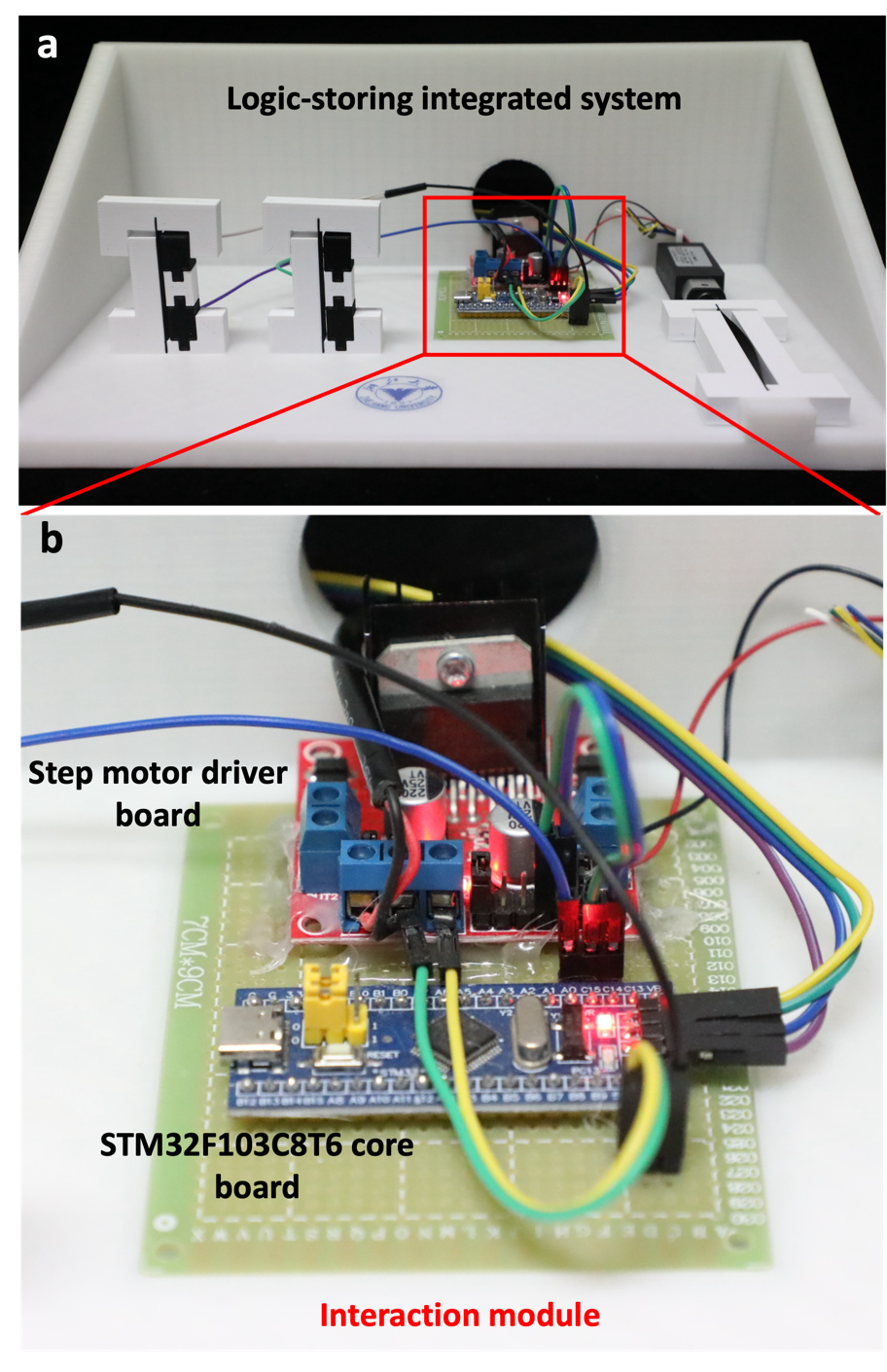


**Fig. S10. Construction of interaction module for logic-storing integrated system. a** Logic-storing integrated system. **b** Interaction module consisting of the core board and step motor driver board.

# Supplementary Note 9: Physical demonstrations of storing Fourier factors by mechanical memories.

Fig. S11 presents the physical demonstrations of storing the results from the fourth-order ODE solvers (i.e., Fourier factors extracted via MATLAB). In particular, a 4×8 storage matrix is used to store the factors of the first-order Fourier series (Fig. S11a), while a 6×8 storage matrix is used to storing the factors of the second-order Fourier series (Fig. S11b).


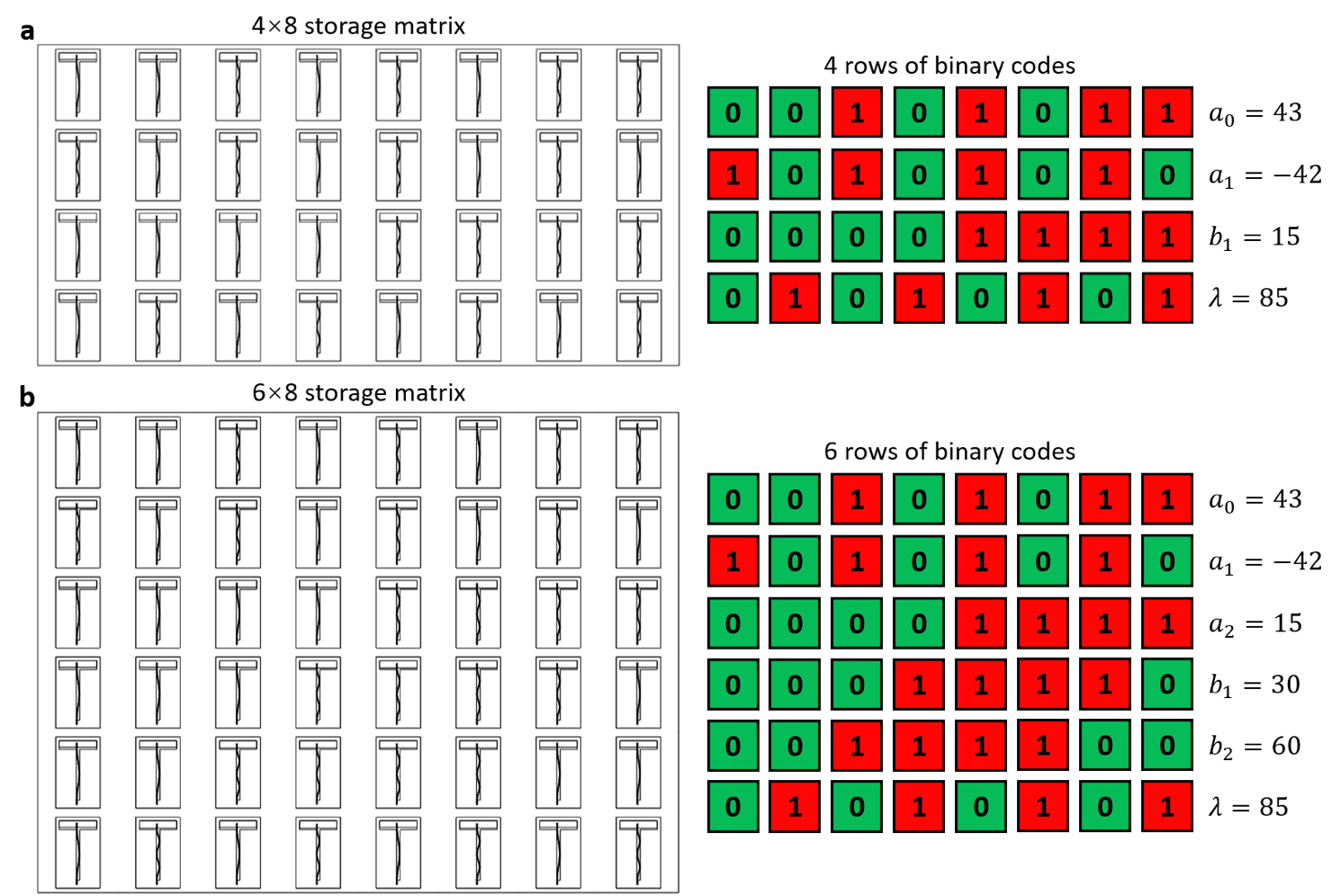


**Fig. S11. Physical demonstrations of storing Fourier factors by mechanical memories. a** Storage of the factors of the first-order Fourier series by a 4×8 storage matrix. **b** Storage of the factors of the second-order Fourier series by a 6×8 storage matrix.

**Movie S1. Experimental test of the multi-instability of bi-walled elastic beam**

**Movie S2. Experimental tests of the fourth-order ODE solvers**

**Movie S3. Experimental demonstrations of the digital logic gates**

**Movie S4. Experimental demonstrations of the mechanical memories**

**Movie S5. Experimental demonstrations of the logic-storing integrated system**

# References

1. Borchani, W., Lajnef, N. & Burgueño, R. Energy method solution for the postbuckling response of an axially loaded bilaterally constrained beam. *Mech. Res. Commun.* **70**, 114-119 (2015).
2. Jiao, P., Borchani, W., Hasni, H., Alavi, A. H. & Lajnef, N. Post-buckling response of non-uniform cross-section bilaterally constrained beams. *Mech. Res. Commun.* **78**, 42-50 (2016).
3. Jiao, P., Yang, Y., Egbe, K. I., He, Z. & Lin, Y. Mechanical metamaterials gyro-structure piezoelectric nanogenerators for energy harvesting under quasi-static excitations in ocean engineering. *ACS Omega* **6**, 15348-15360 (2021).
